# Stochasticity and probabilistic trajectory scoring are essential for data-driven closures of chaotic systems

**DOI:** 10.1073/pnas.2609143123

**Published:** 2026-08-24

**Authors:** Martin T. Brolly

**Affiliations:** ^a^ School of Mathematics, Maxwell Institute for Mathematical Sciences, University of Edinburgh, Edinburgh EH9 3FD, United Kingdom

**Keywords:** stochastic closure, coarse-graining, reduced-order modeling, turbulence, probabilistic forecasting

## Abstract

Coarse-grained models of chaotic systems neglect unresolved degrees of freedom, inducing structured model error that limits predictability and distorts long-term statistics. Typical data-driven closures are trained to minimize prediction error over a single time step, implicitly assuming Markovian dynamics and often failing to capture long-term behavior. Recent approaches instead optimize losses over finite trajectories. However, when such trajectory-based training is carried out with deterministic pointwise losses, it introduces a fundamental mathematical degeneracy. We prove that optimizing pointwise deterministic losses, including but not limited to mean squared error, over chaotic trajectories suppresses predictive variance, with a corresponding loss of physical variability in long integrations. In contrast, strictly proper scoring rules avoid this degeneracy. By targeting forecast distributions rather than realized trajectories, they remove the penalty against predictive spread and align the long-lead optimum with the invariant measure. Using quasi-geostrophic turbulence as a canonical chaotic system, we validate this theory: Closures trained with one-step losses fail to capture stable coarse-grained dynamics, while deterministic closures optimized over trajectories exhibit the variance-loss tendency predicted by our analysis. Stochastic closures calibrated over trajectories using the energy score, however, overcome both structural limitations, yielding skillful ensemble forecasts and realistic long-term statistics. Our results establish that both stochastic modeling and trajectory-based calibration are essential for faithfully representing the dynamics of coarse-grained systems.

## Significance

Simulating complex systems—whether in climate science, biology, or engineering—often requires ignoring components to make computations feasible. But this simplification introduces errors that accumulate over time, causing models to drift away from reality. Conventional data-driven models try to correct this by minimizing one-step prediction errors, which often fails to capture long-term dynamics. Alternatively, optimizing deterministic models over multistep trajectories improves stability but, as we prove, forces a loss of natural variability. We show that faithfully representing effective dynamics requires both trajectory-based calibration and probabilistic modeling. For coarse-grained chaotic systems, randomness is not just useful for uncertainty quantification; it is structurally required for skillful forecasts and realistic long-term behavior.

Many complex systems in science and engineering involve more dynamically relevant degrees of freedom than can be feasibly resolved. Practical models therefore evolve only a subset of variables while treating the remainder implicitly. As a result, they are necessarily coarse-grained: They retain a reduced set of variables while neglecting unresolved components.

This simplification introduces model error with distinctive structure. Unlike measurement noise, the error induced by coarse-graining is dynamical: It arises from deterministic interactions between resolved and unresolved variables. In chaotic systems, where trajectories diverge exponentially and invariant measures govern long-term statistics, even small systematic misrepresentations of these interactions can accumulate, leading to biased forecasts, incorrect variability, and distorted stationary distributions. The resulting discrepancies are not confined to short-term predictive skill; they fundamentally alter the statistical behavior of the model.

The problem of representing unresolved dynamics, commonly referred to as closure or parameterization, has a long history, particularly in fluid dynamics (1) and climate science, where stochastic parameterization has been extensively studied (2–6). Classical closures posit deterministic relationships between the resolved state and the effect of neglected components. More recently, data-driven methods have replaced hand-crafted parameterizations with flexible function approximators trained on high-resolution simulations or observations. These approaches have achieved impressive reductions in one-step prediction error and have demonstrated that machine learning models can flexibly represent how the influence of unresolved dynamics depends on the resolved state.

Most existing data-driven closures are trained in an offline manner: Parameters are optimized to minimize discrepancies between predicted and observed tendencies at a single time step. This procedure implicitly treats the coarse-grained dynamics as Markovian. In complex chaotic systems, this assumption is generally invalid due to the absence of a clear timescale separation between the resolved and unresolved degrees of freedom. For example, in fluid turbulence it has long been recognized that coarse-graining introduces non-Markovian memory (7). While the strength of this non-Markovianity depends on the particular system, it is known to be significant in geophysical turbulence, where the coherence of baroclinic eddies ensures a strong, history-dependent coupling between the resolved and unresolved scales (8, 9). More broadly, in any partially resolved system, the effective dynamics of the resolved variables inevitably inherit both memory and stochasticity from the unresolved components, as formalized in Mori–Zwanzig and related stochastic model-reduction perspectives (10–13). Offline training systematically fails to capture this memory. The accumulation of small one-step biases can severely degrade trajectory statistics, often leading to unphysical bias and/or numerical instability.

It is useful to distinguish three related but separate design choices. First, *memory* concerns the information on which the closure is conditioned: the current resolved state alone, a finite history, previous tendencies, temporally correlated auxiliary variables, or learned latent variables. Second, *stochasticity* concerns whether the closure represents a nondegenerate conditional distribution, rather than a single deterministic prediction, given whatever conditioning information is used. Third, *trajectory-based calibration* concerns the training objective: The model is optimized through its finite-time integrations, rather than only by matching one-step tendencies or transitions. These choices address different aspects of the closure problem. Memory can reduce uncertainty by enriching the conditioning information; stochasticity represents residual uncertainty conditional on that information; and trajectory-based calibration is important because imperfect Markovian or finite-memory closures must be judged by the trajectories they generate when integrated forward.

Building on this distinction, this work studies trajectory-based calibration of coarse-grained closures when the chosen model state does not fully determine the future resolved evolution. To correct temporal errors and implicitly account for missing memory, models are increasingly calibrated over multistep trajectories. We demonstrate that attempting to do this deterministically introduces a fundamental mathematical degeneracy. We prove that when the resolved dynamics are intrinsically stochastic due to coarse-graining, calibration over trajectories using deterministic discrepancy-based losses forces a collapse of the system’s natural variance. Calibration must instead be framed probabilistically; we show that optimizing strictly proper scoring rules avoids this degeneracy, ensuring that the theoretical optimum is consistent with the system’s true conditional law. Crucially, this improvement stems from aligning the learning objective with the stochastic nature of coarse-grained dynamics.

A related phenomenon has been observed in recent data-driven weather forecasting. Deterministic models such as GraphCast (14) and FourCastNet (15) tend to lose fine-scale variability at long lead times. This has motivated growing interest in probabilistic and generative forecasting systems, including GenCast (16) and hybrid models such as NeuralGCM (17). Our results explain why that shift reflects the structure of the problem, rather than merely an ad hoc engineering choice.

We validate these theoretical insights using quasi-geostrophic turbulence as a canonical chaotic system. We show that offline-trained closures become severely miscalibrated over multistep trajectories, often leading to numerical instability, while online-trained deterministic closures suffer the variance collapse predicted by our analysis. In contrast, online-trained probabilistic closures overcome both limitations, yielding skillful ensemble forecasts and realistic stationary spectra. These findings estab- lish that stochasticity and trajectory-based optimization are not independent optional refinements, but coupled, essential mathematical requirements for representing unresolved dynamics in chaotic systems, with implications extending far beyond turbulence modeling.

## 1. The Closure Problem

Consider a dynamical system

[1] $$\begin{array}{c} x_{n+1}=\Phi \left(x_{n}\right). \end{array}$$

Let $\left(\bar{\cdot \;}\right)$ denote a coarse-graining operator, mapping x to a lower-dimensional representation $\bar{x}$, and let $x^{\prime }$ denote the degrees of freedom neglected by $\bar{x}$. In particular, assume there is a function g such that x can be reconstructed from $\bar{x}$ and $x^{\prime }$ via $x=g(\bar{x},\;x^{\prime })$. Suppose we want to construct a model for $\bar{x}$ without solving for $x^{\prime }$. Then we will refer to $\bar{x}$ as the resolved component and $x^{\prime }$ as the unresolved component.

The dynamics of $\bar{x}$ and $x^{\prime }$ are in general coupled, so that we have, for some $\Phi_{1}$ and $\Phi_{2}$,

[2a] $$\begin{array}{cc} {\bar{x}}_{n+1} & =\Phi_{1}\left({\bar{x}}_{n},\;x_{n}^{\prime }\right), \end{array}$$

[2b] $$\begin{array}{cc} x_{n+1}^{\prime } & =\Phi_{2}\left({\bar{x}}_{n},\;x_{n}^{\prime }\right). \end{array}$$

Notice $\Phi_{1}\left({\bar{x}}_{n},\;x_{n}^{\prime }\right)=\bar{\Phi (x_{n})}$.

Now suppose we have a simplified model for the evolution of $\bar{x}$ in the form of a map $\bar{x}\mapsto \bar{\Phi }\left(\bar{x}\right)$. Often a choice of $\bar{\Phi }$ arises from simply neglecting any terms in Φ which depend on $x^{\prime }$. However, we make no assumptions here about $\bar{\Phi }$ or its accuracy. For any $\bar{\Phi }$ we can define the model error

[3] $$\begin{array}{c} m\left({\bar{x}}_{n},\;x_{n}^{\prime }\right)=\Phi_{1}\left({\bar{x}}_{n},\;x_{n}^{\prime }\right)-\bar{\Phi }\left({\bar{x}}_{n}\right). \end{array}$$

Correspondingly, we have

[4] $$\begin{array}{c} {\bar{x}}_{n+1}=\bar{\Phi }\left({\bar{x}}_{n}\right)+m\left({\bar{x}}_{n},\;x_{n}^{\prime }\right). \end{array}$$

The model error accounts for the influence of $x_{n}^{\prime }$ on the evolution of ${\bar{x}}_{n}$, which is not accounted for by $\bar{\Phi }$, as well as any other error introduced by $\bar{\Phi }$.

In order to account for the effect of the unresolved dynamics (i.e. the dynamics of $x^{\prime }$), we must somehow approximate or estimate the model error and form a parameterized model

[5] $$\begin{array}{c} {\tilde{x}}_{n+1}=\bar{\Phi }\left({\tilde{x}}_{n}\right)+{\tilde{m}}_{n}. \end{array}$$

This very general problem is ubiquitous across science and engineering, where employing a truly complete model is impractical. It is known variously as either parameterization or closure.

In classical approaches deterministic approximations to the model error are hypothesized in the form

[6] $$\begin{array}{c} m\left({\bar{x}}_{n},\;x_{n}^{\prime }\right)\approx {\tilde{m}}_{n}=f\left({\bar{x}}_{n}\right), \end{array}$$

for some function f. In this way deterministic closures (or parameterizations) assume that the model error can be diagnosed with certainty from the instantaneous value of the resolved state alone. Since $\Phi_{1}$ depends also on $x_{n}^{\prime }$, this is clearly not true exactly. Rather ${\bar{x}}_{n}$ provides some limited information about $m_{n}$, but uncertainty remains so long as $x_{n}^{\prime }$ is unknown. Intuitively, given a value of ${\bar{x}}_{n}$, there are generally many compatible (physically realizable) values of $x_{n}^{\prime }$, and each of these values causes $\bar{x}$ to evolve along a different trajectory.

Stochastic closures represent this uncertainty explicitly by modeling the model error (and hence the future of the resolved state, via Eq. **5**) probabilistically. Given a stochastic closure, the uncertainty induced in finite-time predictions by uncertain model error can be quantified by ensemble forecasting. However, the benefit of stochastic closures is not limited to uncertainty quantification as an optional extra beyond deterministic predictions. Rather, as we argue in this work, the probabilistic setting of stochastic closures allows for calibration procedures that are aligned with the inherently uncertain dynamics of the coarse-grained state. Put differently, there is an inherent conflict in trying to fit deterministic models to match the stochastic evolution of coarse-grained chaotic systems.

## 2. Data-Driven Closures

Constructing closures that accurately reflect the uncertain future of the coarse-grained state is challenging. The goal of probabilistic forecasting is to design a stochastic closure such that realizations of the parameterized model Eq. **5** are consistent with the conditional law of future coarse-grained trajectories

[7] $$\begin{array}{c} P({\bar{x}}_{n+1},\;\cdots ,{\bar{x}}_{n+m}|{\bar{x}}_{n}=x^{*}), \end{array}$$

which reflect how likely future trajectories are given a coarse-grained initial condition $x^{*}$. The difficulty is that these conditional probabilities usually do not satisfy assumptions that would simplify their representation in models. In particular, the coarse-grained process $\left\{{\bar{x}}_{n}\right\}$ is generally non-Markovian. This means that a factorization of Eq. **7** into a product of one-step transition probabilities $\prod_{i}P\left({\bar{x}}_{n+i+1}|{\bar{x}}_{n+i}\right)$ is not valid.

### 2.1. Offline vs. Online Learning.

In standard approaches, non-Markovianity is ignored; a model of the one-step transition probability is constructed, and this is sampled iteratively at each time step to produce predictions over multiple time steps. We refer to this approach as *offline learning*. Deterministic closures can be considered a special case, where the one-step transition probability has zero variance. Apart from some recent exceptions, most existing work on data-driven closure modeling has followed the offline-learning framework (18–25).

A general Markovian approximation $\left\{{\tilde{x}}_{n}\right\}$ to the coarse-grained process is determined by its own one-step transition probability $\tilde{P}\left({\tilde{x}}_{n+1}|{\tilde{x}}_{n}=x^{*}\right)$, which is typically different from that of the coarse-grained process. Consider a parametric family of Markov kernels $Q_{\theta }\left({\tilde{x}}_{n+1}|{\tilde{x}}_{n}=x^{*}\right)$ with parameters θ. When the aim of predictions is to approximate multistep conditional probabilities as in Eq. **7**, this involves optimizing an objective which depends on predictions over multiple steps of the parameterized model. This trajectory-based approach is known as *online* or *a posteriori learning* (26–29).

### 2.2. Scoring Rules.

The choice of an objective (or loss function) for calibrating data-driven closures is critical. For deterministic closures, loss functions based on mean squared error (MSE) are most common. However, given that the coarse-grained process is intrinsically stochastic, it is more appropriate to evaluate predictions using scoring rules for probabilistic forecasts (30). This has long been recognized in weather forecasting. An extensive literature now exists developing scoring rules and establishing their properties (30–33). A crucial property of certain scoring rules is strict propriety (32): Strictly proper scoring rules are uniquely optimized (in expectation) by the correct probabilistic forecast. A canonical example is the negative log-likelihood, whose minimization mirrors standard maximum likelihood estimation. However, because of high dimensionality and nonlinearity, the likelihood is usually intractable in closure modeling, and it cannot be easily estimated from model samples. Fortunately, strictly proper scoring rules exist that can be estimated directly from finite samples (34). The *energy score* has emerged as a highly effective choice, since it i) is strictly proper, ii) is applicable to multivariate data, and iii) admits a simple unbiased estimator.

Given a forecast measure ν and an observation y, the energy score is defined

[8] $$\begin{array}{c} S\left(\nu ,\;y\right)=E_{\nu }\|Y-y\|-\frac{1}{2}E_{\nu }\left\|Y-Y^{\prime }\right\|, \end{array}$$

where both Y∼ν and $Y^{\prime }∼\nu$ independently. Intuitively, the first term rewards forecasts that place probability mass near the observation, while the second term rewards spread in the forecast distribution. Given a finite ensemble of S samples from ν, $\left\{y^{\left(1\right)},\;\cdots ,\;y^{\left(S\right)}\right\}$, the estimator

[9] $$\begin{array}{cc} \hat{S}(\left\{y^{\left(s\right)}\right\},\;y) & =\frac{1}{S}\sum_{s=1}^{S}\|y^{\left(s\right)}-y\|\\ & -\frac{1}{2S(S-1)}\sum_{s=1}^{S}\sum_{s^{\prime }=1}^{S}\|y^{\left(s\right)}-y^{\left(s^{\prime }\right)}\|, \end{array}$$

satisfies $E_{\nu }\left[\hat{S}\right]=S$ for any S≥2.

Using $\hat{S}$, we can evaluate the predictions of a stochastic model at a given lead time using S≥2 independent samples. Moreover, the energy score naturally accommodates deterministic models: For a point prediction $\hat{y}$, it reduces to the Euclidean distance $S\left(\hat{y},\;y\right)=\left\|\hat{y}-y\right\|$.

For online learning as described above, and given coarse-grained data in the form ${\left\{{\bar{x}}_{n}\right\}}_{n=0}^{N}$, we define a loss function

[10] $$\begin{array}{c} L(\theta \;;\;{\left\{{\bar{x}}_{n}\right\}}_{n=0}^{N})=\frac{1}{N}\sum_{j=1}^{N/w}\sum_{n=(j-1)w+1}^{\mathrm{jw}}\hat{S}({\left\{{\tilde{x}}_{n}^{\left(s\right)}\right\}}_{s=1}^{S},\;{\bar{x}}_{n}), \end{array}$$

where ${\tilde{x}}_{(j-1)w}^{\left(s\right)}={\bar{x}}_{(j-1)w}$ for j=1,⋯,N/w and for each of the S realizations. Here w denotes the window length measured in coarse-model time steps; when reporting results in hours or days we refer to the corresponding physical duration. For stochastic closures (S≥2), $\hat{S}$ is the unbiased estimator defined in Eq. **9**. For deterministic closures (S=1), we extend this notation by evaluating the true energy score functional S analytically on the exact Dirac measure, yielding the Euclidean distance: $\hat{S}(\left\{{\tilde{x}}_{n}^{\left(1\right)}\right\},\;{\bar{x}}_{n})=\left\|{\tilde{x}}_{n}^{\left(1\right)}-{\bar{x}}_{n}\right\|$.

The online loss Eq. **10** can be applied to any model with parameters θ to assess predictions at all lead times up to a horizon w, which we term the *window length*. In practice, the data time series are partitioned into sequential windows of length w; for each window, the model is initialized with the true observed state and integrated forward. The energy score is calculated at each lead time, and the average across the window yields the loss. Note that Eq. **10** reduces to an offline loss in the case w=1.

### 2.3. Generative Closures.

A stochastic closure must specify a conditional law for the model error given the resolved state. We adopt a generative formulation in which the model error is produced by a deterministic function of the resolved state and an auxiliary random input. Let $\xi_{n}$ denote an independent noise variable with known distribution (e.g., standard Gaussian), and define

[11] $$\begin{array}{c} {\tilde{m}}_{n}=G_{\theta }\left({\tilde{x}}_{n},\;\xi_{n}\right). \end{array}$$

This closure is a conditional generative model: Randomness enters only through $\xi_{n}$, while $G_{\theta }$ is a deterministic function governed by parameters θ. Deterministic closures arise as the special case in which $G_{\theta }$ is independent of $\xi_{n}$.

The primary advantage of coupling generative architectures with sample-based scoring rules is that it bypasses the need for a tractable likelihood. Because the energy score can be evaluated purely from model realizations, we are free to employ highly flexible neural networks to represent the complex conditional distribution of ${\tilde{x}}_{n+1}$. Furthermore, this formulation places deterministic and stochastic closures within a unified mathematical framework, differing only in whether the learned mapping actively depends on the auxiliary noise.

Importantly, this generative approach does not impose Gaussianity, linearity, or additive structure on the model error. The only structural assumption enforced here is Markovianity. Non-Markovianity of the true coarse-grained dynamics is not represented explicitly in this formulation; rather, trajectory-based calibration encourages the Markovian closure to compensate for missing memory over finite horizons.

### 2.4. Asymptotic Properties of Trajectory-Based Objectives.

The core mechanism of online learning is the optimization of an objective function that accumulates loss over a finite time window. Because the parameters θ are shared across steps of the window, a model is optimized when it balances loss across lead times. In chaotic systems with mixing dynamics, however, the true future state ${\bar{x}}_{n+m}$ asymptotically decorrelates from the initial condition ${\bar{x}}_{n}$. As this decorrelation occurs, precise trajectory tracking becomes impossible. Consequently, loss at large lead times is not governed by short-term forecasting skill, but rather by how the objective function evaluates the model’s longer-term predictions against the unpredictable future evolution of the true coarse-grained dynamics. To understand how deterministic learning objectives fail, it is instructive to analyze this asymptotic regime, since even with finite window lengths the imprint of this limit is critical.

We now analyze long-lead training under a common stationary setup. We assume the complete system preserves a probability measure μ on the full state space. We let $\overset{¯}{\mu }$ denote its pushforward onto the coarse-grained space and assume $\overset{¯}{\mu }$ has finite second moment. We assume the coarse-grained initial condition is distributed as $\overset{¯}{\mu }$ and compare the true coarse-grained dynamics with a Markovian model initialized from the exact resolved state and evolved according to

$${\tilde{x}}_{n+k+1}=F_{\theta }\left({\tilde{x}}_{n+k},\;\xi_{n+k}\right),\;{\tilde{x}}_{n}={\bar{x}}_{n},$$

where the noise variables are independent.

The following proposition states the long-lead degeneracy for MSE. This case is useful because the mechanism is especially transparent through a bias–variance decomposition. The restriction to MSE is not essential: A corresponding result for broader classes of pointwise deterministic losses is given in *SI Appendix* and summarized below.

Proposition 1 (Degeneracy of long-lead mean-squared-error training). *Assume further that the coarse-grained state decorrelates under the dynamics in the sense that*

$$\begin{array}{c} E[{\bar{x}}_{n+m}|{\bar{x}}_{n}]\;\overset{L^{2}\left(\overset{¯}{\mu }\right)}{\to }\;E_{\overset{¯}{\mu }}\left[\bar{x}\right]\;\;\mathrm{as}\;m\to \infty . \end{array}$$

*Denote the MSE loss at lead time* m *as*

$$\begin{array}{c} J_{m}\left(\theta \right)=E[{\left\|{\tilde{x}}_{n+m}-{\bar{x}}_{n+m}\right\|}^{2}]. \end{array}$$

*This objective admits a decomposition of the form*

$$\begin{array}{c} \begin{array}{cc} J_{m}(\theta ) & =E[{\left\|E[{\tilde{x}}_{n+m}|{\bar{x}}_{n}]-E[{\bar{x}}_{n+m}|{\bar{x}}_{n}]\right\|}^{2}]\\ & +E[\text{Tr}(\text{Cov}({\tilde{x}}_{n+m}|{\bar{x}}_{n}))]+C_{m}, \end{array} \end{array}$$

*where* $C_{m}$ *is independent of* θ.

*In particular, predictive variance enters the MSE loss as an additive nonnegative term. Under the stated decorrelation assumption, the target conditional mean in the first term approaches the climatological mean at long lead times. Thus, the long-lead contribution to the MSE increasingly favors forecasts with vanishing spread and predictive means close to climatology.*

A detailed proof of this proposition is provided in *SI Appendix*.

The decomposition in *Proposition* 1 highlights the mechanism by which deterministic training over extended windows becomes systematically biased. At each lead time, the MSE contains two parameter-dependent contributions: one penalizing the discrepancy between the model’s conditional mean and the target’s conditional mean and another directly penalizing predictive variance. As lead time increases, the target conditional mean becomes less dependent on the initial state and converges to the constant climatological mean. The long-lead terms in a time-averaged MSE objective therefore increasingly favor forecasts with suppressed spread and means close to climatology. Because the same parameters govern every step of the model integration, this long-lead pressure feeds back onto the learned dynamics, biasing them toward excess dissipation and helping explain the oversmoothing widely observed in deterministic weather and climate emulators.

Informally, once the verifying state is effectively unpredictable from the initial condition, a pointwise deterministic loss rewards forecasts that remain close to a climatological center and penalizes forecast-to-forecast variability, even when that variability is physically appropriate.

#### 2.4.1. Remark on generality.

Although *Proposition* 1 is stated for the MSE, *SI Appendix* shows that the same long-lead collapse holds for any pointwise discrepancy loss, including the Euclidean distance used to train our deterministic closures. In particular, we show that for any pointwise loss induced by a bivariate discrepancy function ℓ(·,·), the asymptotic objective reduces to the expectation of the climatological risk field

$$R\left(\tilde{y}\right):=E_{\bar{y}∼\overset{¯}{\mu }}[\ell \left(\tilde{y},\;\bar{y}\right)].$$

If R has a unique global minimizer $c^{*}$, then the long-lead optimum is attained only when the forecast distribution collapses to the Dirac measure at $c^{*}$. Metric losses are an important special case: When ℓ is a metric, $c^{*}$ is the corresponding Fréchet median of the invariant measure. Proper scoring rules are therefore required not merely to replace MSE, but to avoid the broader failure of pointwise trajectory matching.

In contrast to the systematic loss of variance induced by pointwise deterministic training, strictly proper scoring rules remove this structural bias. Assume now that the true lead-time conditional distributions converge to the invariant measure, and that the model’s predictive distributions admit a well-defined long-lead limit. *Proposition* 2 shows that, under the continuity and integrability assumptions stated in *SI Appendix*, the asymptotic expected score decomposes into a constant depending only on the invariant measure and the scoring rule, plus the expected divergence induced by the scoring rule between the model’s long-lead predictive distributions and $\overset{¯}{\mu }$. Thus probabilistic trajectory-based training does not penalize predictive spread in itself; it penalizes only distributional mismatch with the true invariant measure.

To state the result, let $P_{2}$ denote the set of Borel probability measures on the coarse-grained space with finite second moment. For each lead time m, let $Q_{m}\left(\;\cdot |{\bar{x}}_{n}\right)$ denote the true lead-time conditional law, let ${\tilde{P}}_{\theta ,m}\left(\;\cdot |{\bar{x}}_{n}\right)$ denote the model’s m-step predictive law, and define the corresponding expected score by

$$\begin{array}{c} L_{m}\left(\theta \right):=E_{{\bar{x}}_{n}}[E_{\bar{y}∼Q_{m}\left(\;\cdot \;|{\bar{x}}_{n}\right)}[S({\tilde{P}}_{\theta ,m}\left(\;\cdot |{\bar{x}}_{n}\right),\;\bar{y})]]. \end{array}$$

Proposition 2 (Asymptotic consistency of strictly proper scoring rules). *Under the common stationary setup above, let* S(P,y) *be a strictly proper scoring rule on* $P_{2}$. *Assume additionally that, for* $\overset{¯}{\mu }$*-almost every resolved initial condition* ${\bar{x}}_{n}$,

$$\begin{array}{c} Q_{m}\left(\;\cdot |{\bar{x}}_{n}\right)\overset{W_{2}}{\to }\overset{¯}{\mu }\left(\;\cdot \;\right) \end{array}$$

*as* m→∞, *and that the model predictive laws admit a* $W_{2}$ *limit, denoted by* ${\tilde{P}}_{\theta ,\infty }\left(\;\cdot |{\bar{x}}_{n}\right)$, *in the sense that*

$$\begin{array}{c} {\tilde{P}}_{\theta ,m}\left(\;\cdot |{\bar{x}}_{n}\right)\overset{W_{2}}{\to }{\tilde{P}}_{\theta ,\infty }\left(\;\cdot |{\bar{x}}_{n}\right). \end{array}$$

*Assume also that the continuity and integrability assumptions stated in* *SI Appendix* *hold*.

*Then*

$$\begin{array}{c} \lim_{m\to \infty }L_{m}\left(\theta \right)=E_{{\bar{x}}_{n}}[d_{S}({\tilde{P}}_{\theta ,\infty }\left(\;\cdot |{\bar{x}}_{n}\right),\;\overset{¯}{\mu })]+E_{\bar{y}∼\overset{¯}{\mu }}[S\left(\overset{¯}{\mu },\;\bar{y}\right)], \end{array}$$

*where* $d_{S}$ *denotes the divergence induced by* S. *onsequently, the parameter-dependent part of the asymptotic objective is exactly the expected divergence between the model’s long-lead predictive law and the invariant measure.*

Here

$$\begin{array}{c} d_{S}\left(P,\;Q\right):=E_{y∼Q}[S\left(P,\;y\right)]-E_{y∼Q}[S\left(Q,\;y\right)]\geq 0, \end{array}$$

with equality if and only if P=Q. The second term in *Proposition* 2 is therefore the generalized entropy induced by S evaluated at the invariant measure and is independent of the model parameters.

Informally, once the future state is no longer predictable from the initial condition, a proper score rewards the model for producing the correct distribution of possible states, rather than for staying close to a single representative state. At long lead times, this correct distribution is the invariant measure.

Convergence in the 2-Wasserstein metric ($W_{2}$) combines weak convergence with convergence of second moments. From a physical perspective, this means that agreement at the optimum is not limited to weak distributional agreement, but extends to quadratic quantities such as variance and kinetic energy. If the divergence term in *Proposition* 2 vanishes, then the model’s long-lead predictive distribution coincides with $\overset{¯}{\mu }$, and its covariance matches the climatological covariance ${\text{Cov}}_{\overset{¯}{\mu }}\left(\bar{x}\right)$. More generally, for parametric models, the parameter-dependent part of the asymptotic objective is exactly the expected divergence from $\overset{¯}{\mu }$. Minimizing a strictly proper score therefore drives the model’s asymptotic distribution as close as the model class allows to the true invariant measure, rather than toward a variance-collapsed deterministic climatology.

A detailed proof is provided in *SI Appendix*.

## 3. Numerical Results

**Fig. 1.**
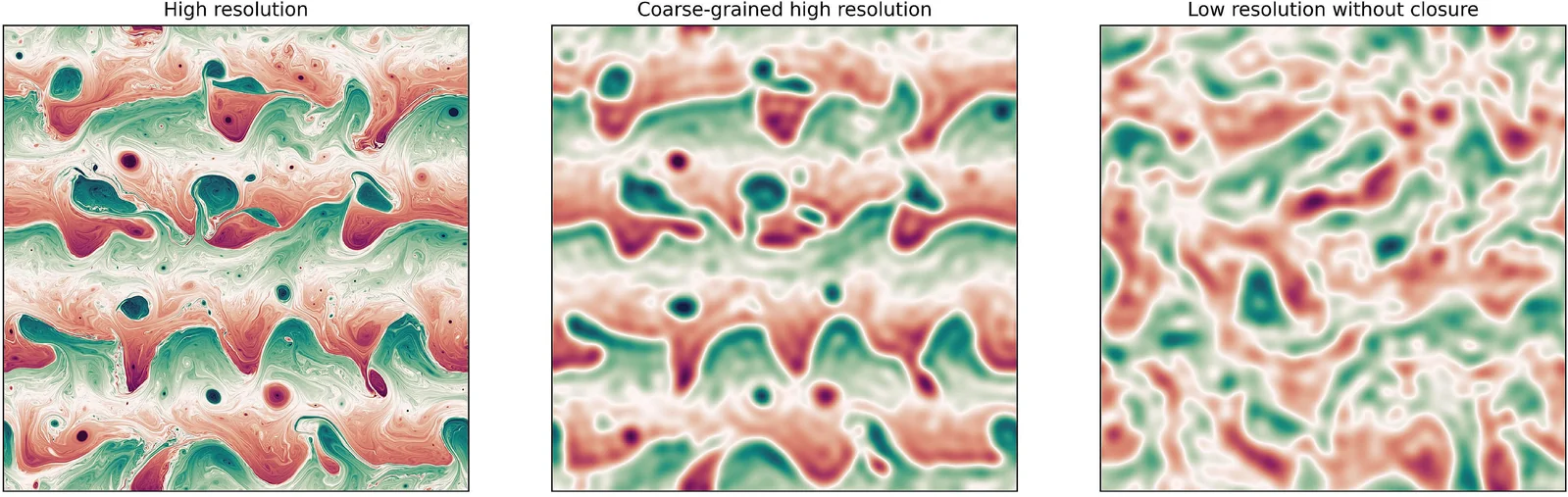
Snapshots of upper-layer potential vorticity anomaly in the quasi-geostrophic model at high resolution (*Left*), after coarse-graining (*Center*), and from a free-running low-resolution simulation without parameterization (*Right*).

We now demonstrate the critical role of stochasticity and online learning in data-driven closure of quasi-geostrophic (QG) turbulence. QG turbulence, developed as an idealized model of large-scale atmosphere/ocean dynamics, is a canonical example of a high-dimensional nonlinear physical system, governed by an active scalar transport-type partial differential equation. Fig. 1 shows snapshots of potential vorticity anomaly in the QG model i) at high resolution (which we consider the complete model system, corresponding to Eq. **1**), ii) after applying a coarse-graining operator (corresponding to the coarse-grained dynamics Eq. **2a**), and iii) from an equivalent low-resolution simulation without closure (corresponding to Eq. **5** with $\tilde{m}\equiv 0$). The dynamics and statistics of the low-resolution model are markedly different from those of the coarse-grained system. We are interested in learning closures from coarse-grained simulations to reduce this discrepancy.

**Fig. 2.**
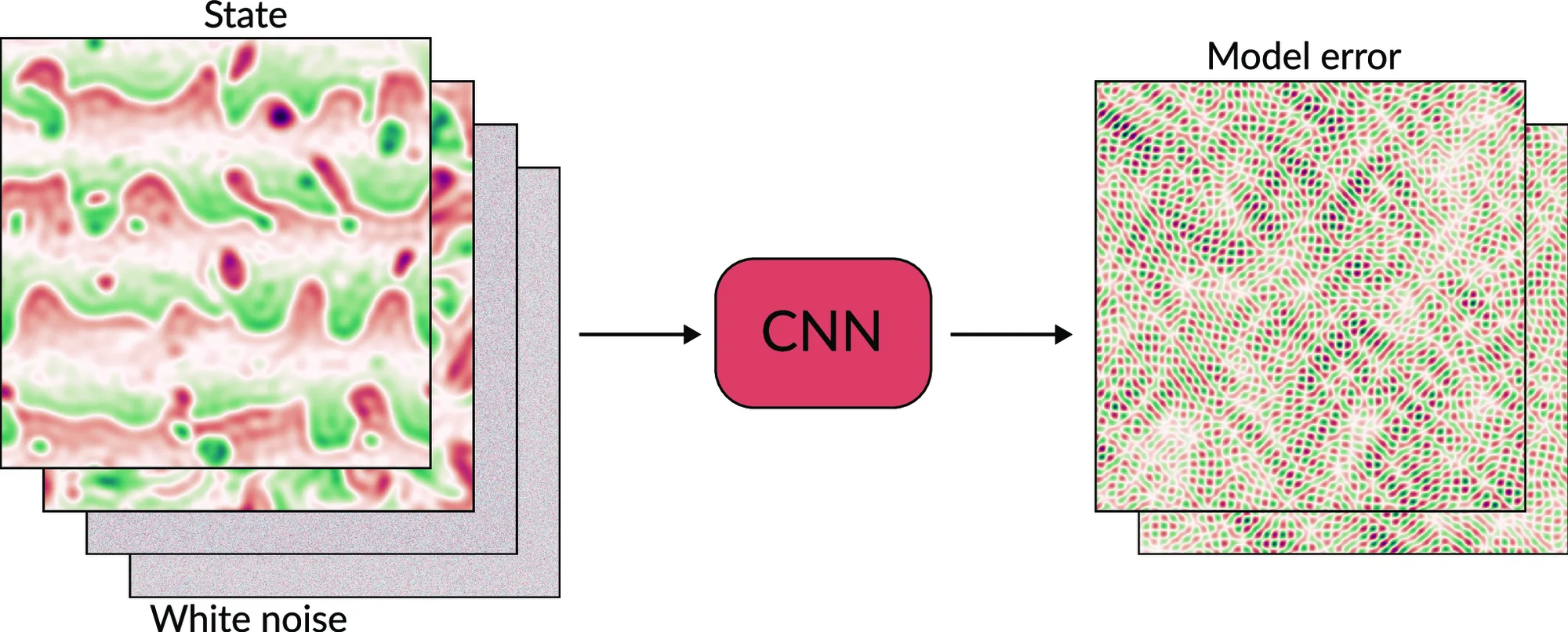
Schematic of the generative closures used in the quasi-geostrophic turbulence experiments. This is an example of Eq. **11**, where $G_{\theta }$ is a convolutional neural network, ${\bar{x}}_{n}$ is the instantaneous potential vorticity anomaly, and ξ is a random field where $\text{dim}\xi_{n}=\text{dim}{\bar{x}}_{n}$ and each component is an independent standard Gaussian random variable.

In order to avoid conflation with other approximation/ estimation issues, we employ highly flexible generative parameterizations of the form Eq. **11** with $G_{\theta }$ given by a deep convolutional neural network (Fig. 2), and use a large amount of data (100 y of coarse-grained simulation data) to optimize the network parameters θ with respect to the loss Eq. **10**.

We train both deterministic and stochastic closures in this way with various window lengths (w in Eq. **10**). Deterministic closures are constructed straightforwardly by training in the same way but without the random input $\xi_{n}$ in Eq. **11** and setting S=1, thereby explicitly optimizing the deterministic Euclidean distance reduction of the energy score. Because the stochastic architecture is structurally identical to this deterministic baseline—differing only by the inclusion of the auxiliary noise field—the significant improvements demonstrated below are attributable solely to the probabilistic training objective rather than added architectural complexity.

The CNN closure used here is spatially nonlocal but temporally Markovian. Spatial nonlocality means that the predicted model error at a grid point can depend on the value of the instantaneous resolved field at other points in space. This is distinct from temporal memory, which retains information from previous resolved states or auxiliary variables. The present CNN therefore learns a state-dependent spatial mapping from the instantaneous resolved field while remaining within a temporally Markovian closure class.

In the following sections we evaluate the parameterized models using an additional 100 y of simulation data not used in training. Full details of the QG model, the coarse-graining operator, and the training procedure are given in *SI Appendix*.

### 3.1. Finite-Time Predictive Skill.

**Fig. 3.**
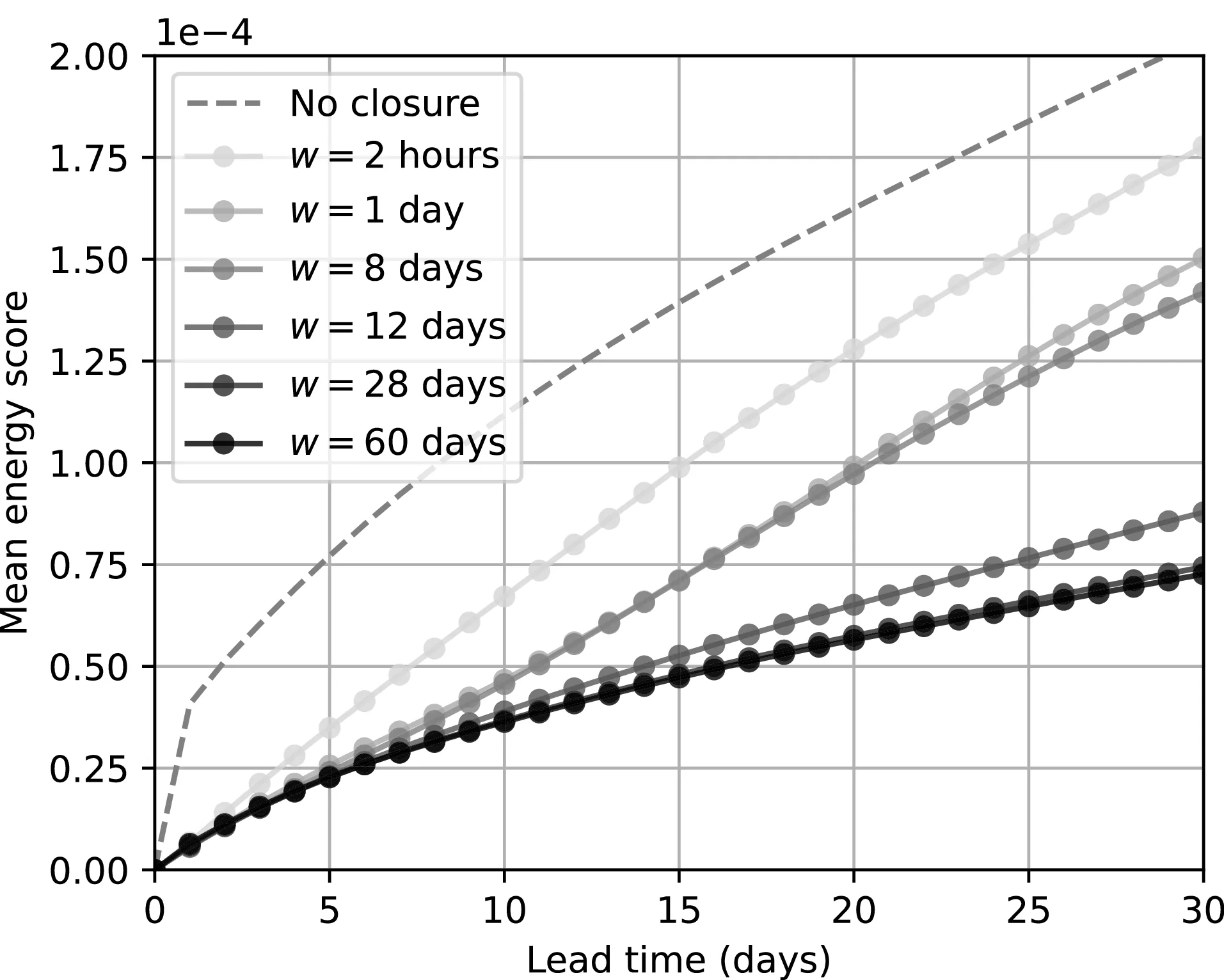
Mean energy score as a function of lead time for stochastic closures trained with various window lengths w. Increasing window length improves forecast skill consistently across lead times until a plateau is reached at w on the order of several weeks.

We evaluate the finite-time predictive skill of the parameterized models by computing the mean energy score on the validation data. The validation data are partitioned into 30-d windows. For each window, 20 realizations of each parameterized model are initialized from the true state, and the energy score estimator in Eq. **9** is computed at lead times from 1 to 30 d. The resulting scores are averaged over the collection of windows. Fig. 3 shows these mean energy scores for the stochastic closures for a range of window lengths w. In particular, w=2 h corresponds to a single time step, and therefore offline learning. As w is increased mean energy scores are seen to improve (decrease) across a range of lead times, until at window lengths on the order of several weeks (hundreds of model time steps) the performance plateaus.

**Fig. 4.**
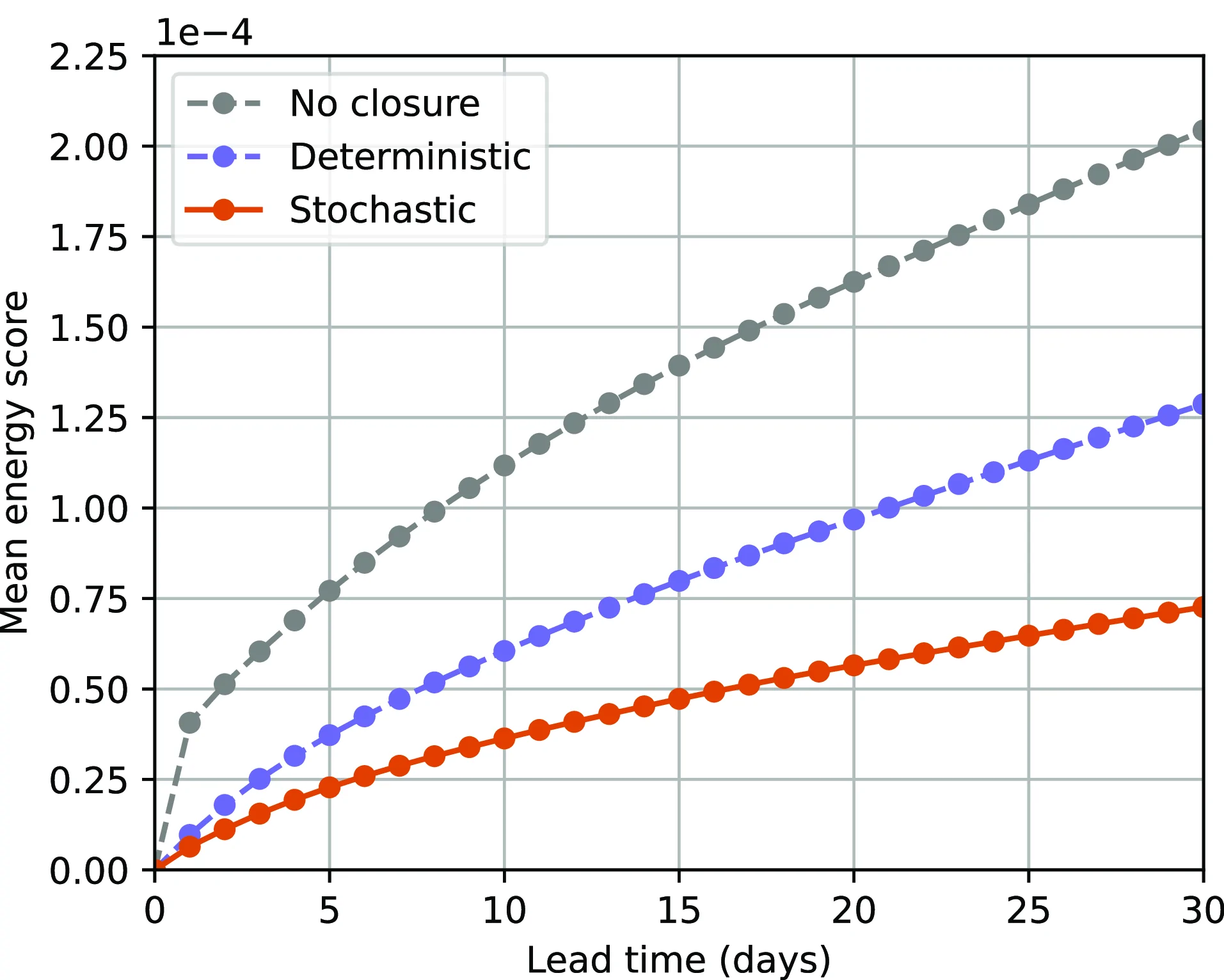
Mean energy score as a function of lead time for online-trained deterministic and stochastic closures, as well as for the coarse model without a closure. Both closures improve on the unparameterized coarse model, but the stochastic closure gives consistently lower energy scores than the deterministic closure.

In Fig. 4 mean energy scores are shown for deterministic and stochastic closures, as well as for the coarse model without closure. In particular, the scores shown correspond to closures trained with the value of w which optimizes long-term error in the kinetic energy spectrum, as discussed in the next section. While both deterministic and stochastic closures lead to improvements in forecasting skill, the stochastically parameterized models outperform the deterministic models significantly at all lead times considered.

### 3.2. Stationary Statistics.

We now consider the long-term consistency of the parameterized models with the coarse-grained dynamics by comparing time-averaged kinetic energy spectra. In particular, we consider the depth-averaged isotropic kinetic energy spectrum, which we denote $\bar{E}\left(\kappa \right)$ for the true coarse-grained dynamics and $\tilde{E}\left(\kappa \right)$ in the case of a parameterized model, with $\kappa^{2}=k_{x}^{2}+k_{y}^{2}$. We define an error between the two as

[12] $$\begin{array}{c} \Delta E=\frac{1}{\kappa_{\text{c}}}\int_{0}^{\kappa_{\text{c}}}\;{\left[\log\frac{\tilde{E}\left(\kappa \right)}{\bar{E}\left(\kappa \right)}\right]}^{2}\;d\kappa , \end{array}$$

where $\kappa_{\text{c}}=\frac{2}{3}k_{\text{max}}$ is a cut-off wavenumber. This definition is dimensionless and chosen to be sensitive to errors across a range of wavenumbers.

**Fig. 5.**
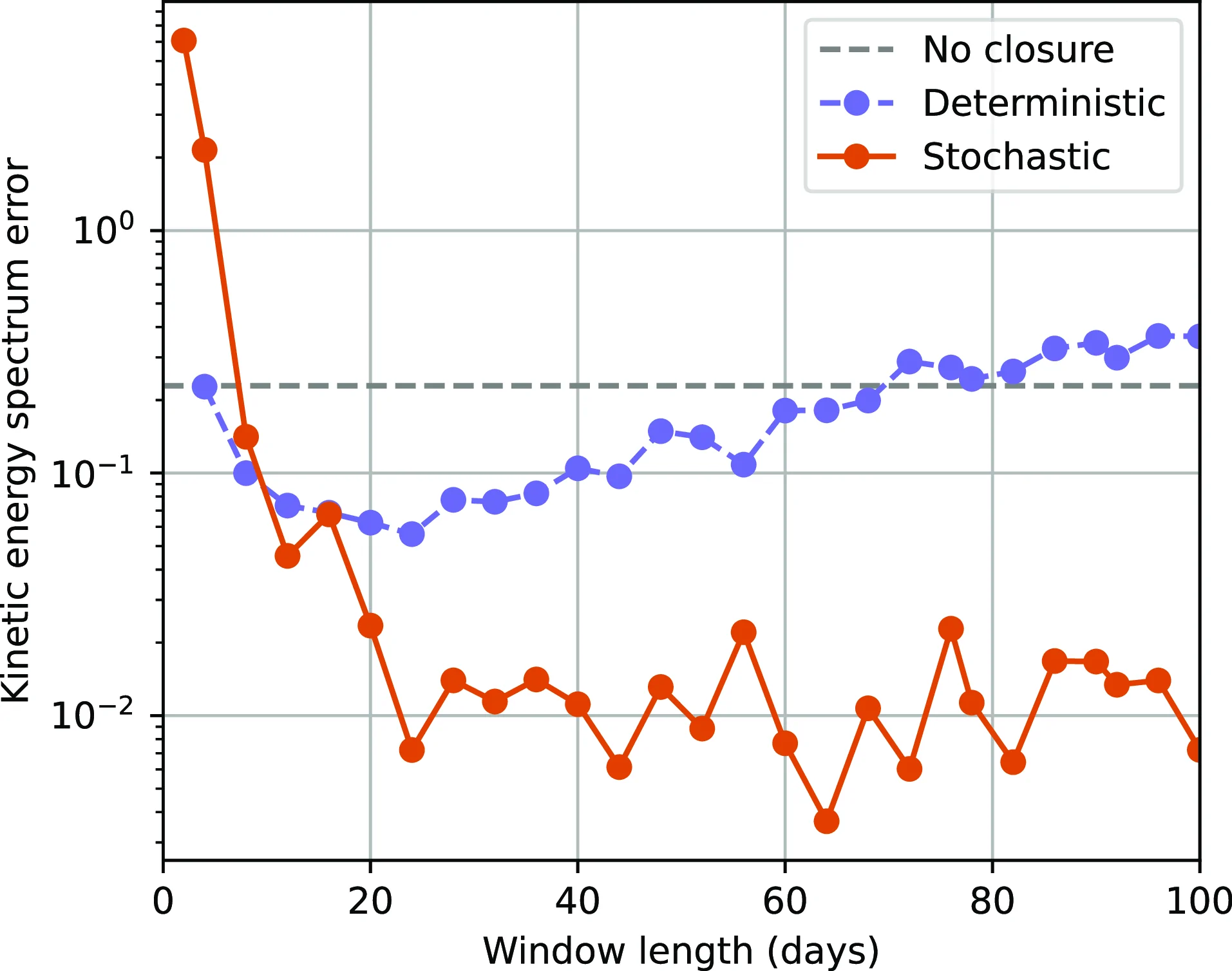
Errors in kinetic energy spectrum as a function of training window length w, for deterministic and stochastic closures, as well as without closure. Deterministic closures show an intermediate optimum before degrading at longer windows, whereas stochastic closures continue to improve before reaching a broad plateau.

Fig. 5 shows ΔE as a function of window length for both deterministic and stochastic closures. The error is also shown for the coarse model without closure for reference. In order to compute these errors, the coarse model was run with each closure for 100 y. For deterministic closures, window lengths shorter than 4 d produced models that became unstable before the integrations were completed. For stochastic closures, the same occurred for window lengths shorter than 2 d, including the offline model at w=2 h. These cases appear as missing points in Fig. 5, since ΔE could not be computed once the simulations became unstable. Thus, stochasticity alone is not sufficient to stabilize short-window state-only closures: Sufficiently long trajectory-based calibration is still required to accommodate the temporal mismatch between the Markovian closure and the coarse-grained dynamics.

For deterministic closures, an optimal window length appears at an intermediate value of about w=24 d, where ΔE is noticeably improved relative to the coarse model without closure. This intermediate optimum reflects the compromise imposed by the temporally averaged pointwise loss. At short windows, the model is driven to track realized sample trajectories. As the window is lengthened, the long-lead terms highlighted by the SI generalization of *Proposition* 1 increasingly penalize predictive spread and deviations from climatology. In the present setting of QG turbulence, much of the variability being averaged over is concentrated at relatively small scales, where unresolved fluctuations are strongest. The deterministic model is therefore pushed toward a conditional average that preferentially damps those rapidly varying components. Because the same parameters govern every lead time in the window, this long-lead pressure feeds back onto the learned dynamics as a whole, producing excess dissipation and the overly smooth states observed for larger window lengths.

This issue of oversmoothing has been widely observed in the emulation of weather models (14, 35–37) and in parameterization in atmospheric and oceanic modeling (17, 29). As established in *SI Appendix*, this phenomenon is a direct consequence of the degeneracy of any pointwise loss at long lead times, which forces the model to collapse its predictive variance. Spectral losses can mitigate some symptoms of this problem by directly penalizing errors in Fourier coefficients, spectral amplitudes, or derived spectra, but since they are typically implemented as pointwise losses, they do not remove the long-lead collapse mechanism. In contrast, *Proposition* 2 shows that strictly proper scoring rules remove this long-lead bias against predictive spread by aligning the asymptotic objective with the invariant measure.

Consistent with this, once the training window is long enough to stabilize the model, the stochastic closures show a continued reduction in ΔE as w is increased, before approaching a broad plateau at the largest window lengths considered. The noise visible at the longest window lengths is practical rather than structural: As w grows, the fixed 100-y training record contains proportionally fewer disjoint trajectories of length w, making the online optimization noisier.

**Fig. 6.**
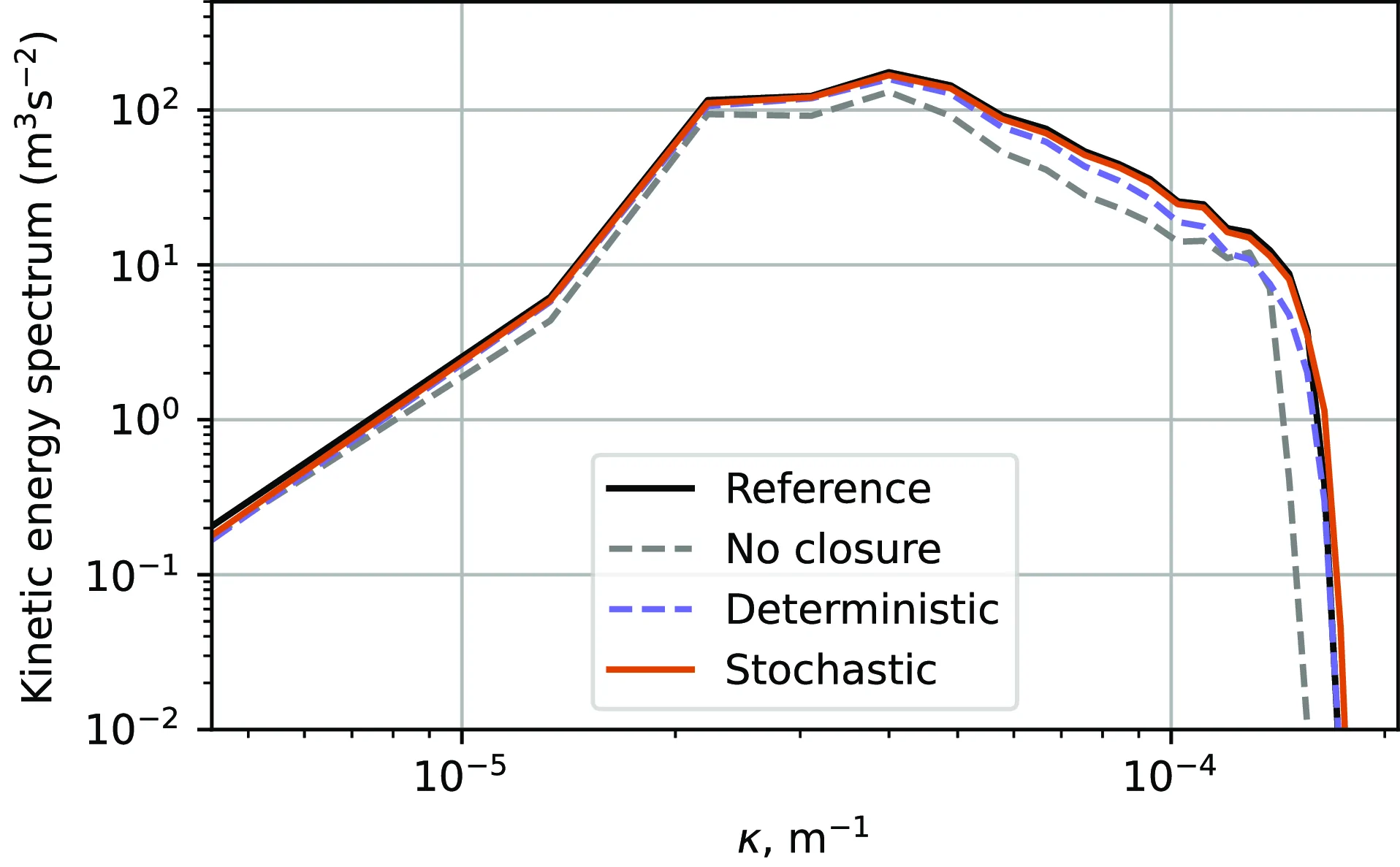
Height-averaged isotropic kinetic energy spectra. Spectra are shown for the true coarse-grained system, the coarse model without closure ($\Delta E=2.29\times 10^{-1}$), and the deterministic ($\Delta E=5.60\times 10^{-2}$) and stochastic ($\Delta E=3.67\times 10^{-3}$) closures. For both closures, results correspond to the window length w yielding the lowest spectrum error. The deterministic closure underestimates small-scale energy, while the stochastic closure closely matches the coarse-grained spectrum.

In Fig. 6 we show the kinetic energy spectrum for the true coarse-grained system, the coarse model without closure, and with deterministic and stochastic closures. For each closure we show the spectrum corresponding to the window length w yielding the lowest spectrum error ΔE. Even with this optimally tuned w, the deterministic closure is unable to recover the kinetic energy spectrum accurately, exhibiting a substantial, artificial decrease in energy at scales smaller than approximately 25km. In contrast, the stochastic closure achieves the correct distribution of energy down to the dissipation scale.

**Fig. 7.**
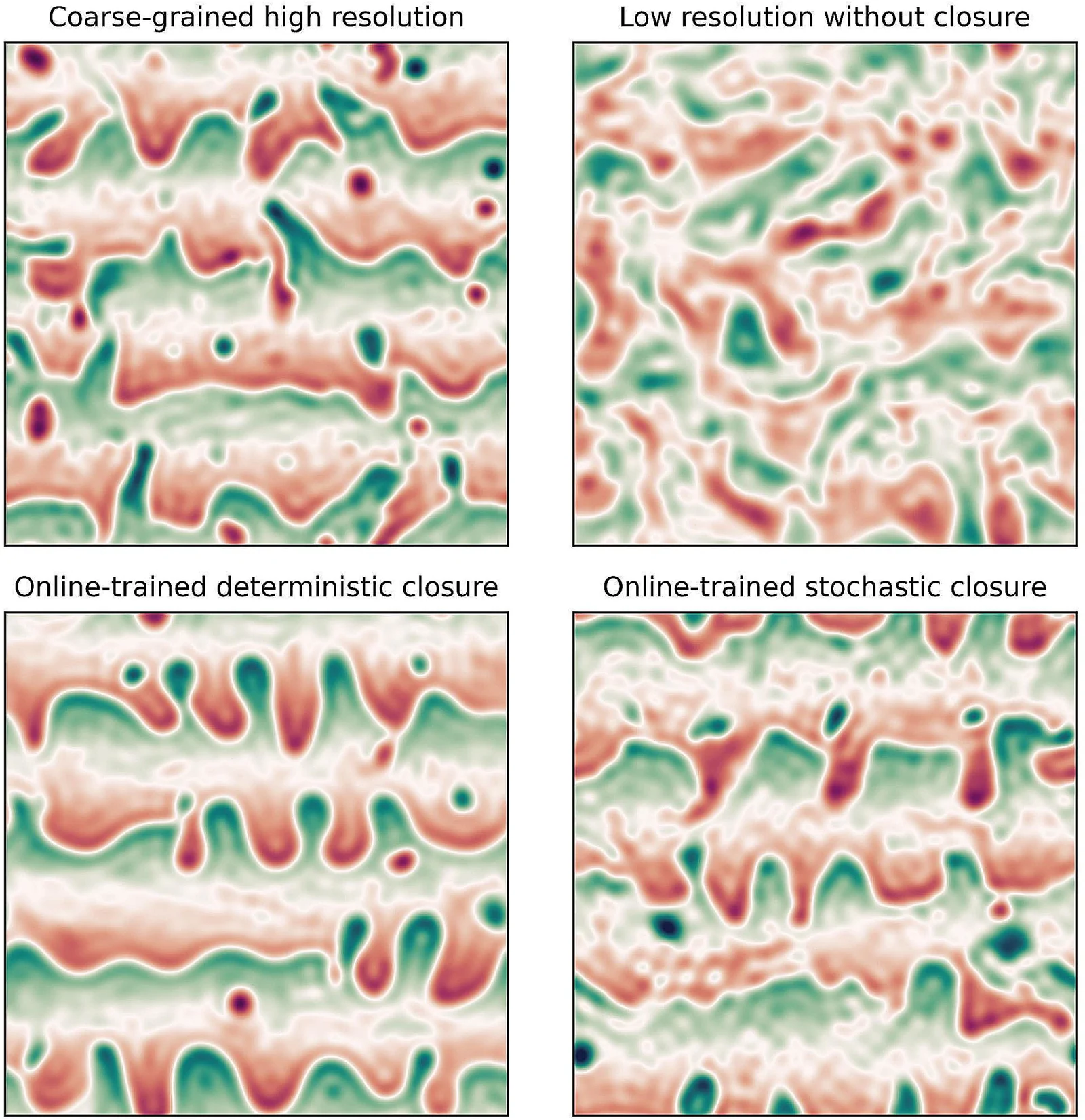
Snapshots of upper-layer PV anomaly from free-running simulations after 100 y with the best online-trained closures, both stochastic and deterministic. Offline-trained closures are omitted because they became unstable before the end of the integrations. The deterministic closure produces an overly smooth state, whereas the stochastic closure maintains coherent structures and small-scale variability closer to the true coarse-grained dynamics.

To complement the spectral analysis, Fig. 7 visualizes snapshots of upper-layer PV anomaly fields after 100 y of simulation with the best online-trained deterministic and stochastic closures, as well as in the true coarse-grained system and at low resolution without any closure. Offline-trained deterministic and stochastic closures are omitted because both became unstable before the end of the 100-y integrations. Note first the difference between the target process $\left({\bar{x}}_{n}\right)$ and the unparameterized coarse simulation [corresponding to ${\tilde{x}}_{n+1}=\bar{\Phi }\left({\tilde{x}}_{n}\right)$]: The coherent structure of jets and eddies is lost at low resolution without closure. The online-trained deterministic closure led to overly smooth fields, a direct physical manifestation of the variance-loss tendency described by *Proposition* 1. By contrast, the online-trained stochastic closure sustains a physically realistic flow field that is visually consistent with the true coarse-grained dynamics. This comparison encapsulates the two structural limitations identified above: Short-window state-only training fails to accommodate unresolved memory, while deterministic pointwise trajectory training suppresses natural variability. Probabilistic trajectory-based calibration avoids both pathologies and maintains both large-scale coherent structures and small-scale turbulent fluctuations.

## 4. Conclusions

The development of numerical models of complex systems inevitably involves neglecting certain degrees of freedom. A standard approach to calibrating such models is to minimize one-step deterministic prediction errors, such as the MSE. Although evaluating deterministic metrics over extended trajectories is becoming more common, with the aim of improving stability, our analysis demonstrates that doing so introduces a systematic long-lead bias. As established analytically and validated numerically, optimizing any deterministic distance metric over multistep trajectories inherently forces the model to damp out natural variability, leading to a collapse of predictive variance and distorted long-term statistics.

To represent the dynamics of coarse-grained systems accurately, model calibration must account for the intrinsic uncertainty induced by unresolved components. We have shown that when models are formulated probabilistically and tuned over finite trajectories using strictly proper scoring rules, this structural degeneracy is avoided. Trajectory-based probabilistic scoring aligns the calibration objective with the true dynamics, allowing models to simultaneously capture short-term conditional evolution and the correct long-term invariant measure.

Importantly, these findings extend beyond machine learning parameterizations in fluid dynamics, applying generally to the calibration of any reduced-order model. Whenever a chaotic system is only partially resolved, standard deterministic calibration will predictably degrade long-term fidelity. Trajectory-based probabilistic learning is therefore not merely an optional add-on for uncertainty quantification, but an essential ingredient in building statistically faithful reduced models of coarse-grained systems.

## 5. Materials and Methods

High-resolution data were generated from a two-layer quasi-geostrophic turbulence model on a doubly periodic domain. In the learning problem, the state at time $t_{n}$ was the stacked pair of layerwise potential-vorticity-anomaly fields: The full state $x_{n}$ was defined on the fine grid, and the resolved state ${\bar{x}}_{n}$ was obtained by coarse-graining to the low-resolution grid. The coarse-graining operator combined spectral truncation with a low-pass filter. After an initial spin-up to approximate stationarity, 100 y of simulation output were used for training and an independent 100-y dataset was used for validation and evaluation.

Closures were represented as conditional generative models for unresolved model error,

$$\begin{array}{c} {\tilde{m}}_{n}=G_{\theta }\left({\tilde{x}}_{n},\;\xi_{n}\right), \end{array}$$

where $G_{\theta }$ was a convolutional neural network and $\xi_{n}$ was an independent standard Gaussian noise field. Deterministic closures were obtained by removing the noise input. Models were trained either offline with one-step losses or online over finite windows. Stochastic closures were calibrated with the energy score, estimated from model ensembles, and optimization used AdamW with a curriculum in window length. Predictive skill was evaluated using validation energy scores, and long-run fidelity was assessed using time-averaged kinetic-energy spectra from long free-running simulations. Full equations, parameter values, architecture details, and numerical methods are given in *SI Appendix*.

## Supplementary Material

Appendix 01 (PDF)

## Data Availability

The JAX implementation of the quasi-geostrophic model and all other code required to reproduce the results herein are available on Zenodo (38). The trained neural network models are also available on Zenodo (39).

## References

[1] S. B. Pope, Turbulent Flows (Cambridge University Press, 2000).

[2] D. S. Wilks, Effects of stochastic parametrizations in the Lorenz ’96 system. Q. J. R. Meteorol. Soc. **131**, 389–407 (2005).

[3] A. J. Majda, I. Timofeyev, E. Vanden Eijnden, A mathematical framework for stochastic climate models. Commun. Pure Appl. Math. **54**, 891–974 (2001).

[4] A. Majda, I. Timofeyev, E. Vanden-Eijnden, Stochastic models for selected slow variables in large deterministic systems. Nonlinearity **19**, 769–794 (2006).

[5] H. Christensen, L. Zanna, “Parametrization in weather and climate models” in *Climate Science, Oxford Research Encyclopedias*, H. V. Storch, Ed. (Oxford University Press, 2022). vol. 12.

[6] J. Berner , Stochastic parameterization: Toward a new view of weather and climate models. Bull. Am. Meteorol. Soc. **98**, 565–588 (2017).

[7] R. H. Kraichnan, The structure of isotropic turbulence at very high Reynolds numbers. J. Fluid Mech. **5**, 497–543 (1959).

[8] H. A. Dijkstra, G. Manucharyan, W. Moon, Eddy memory in weakly nonlinear two-layer quasi-geostrophic ocean flows. Eur. Phys. J. Plus **137**, 1162 (2022).

[9] E. Vanderborght, J. Demaeyer, G. Manucharyan, W. Moon, H. A. Dijkstra, Physics of the eddy memory kernel of a baroclinic midlatitude atmosphere. J. Atmos. Sci. **81**, 691–711 (2024).

[10] A. J. Chorin, O. H. Hald, R. Kupferman, Optimal prediction and the Mori-Zwanzig representation of irreversible processes. Proc. Natl. Acad. Sci. U.S.A. **97**, 2968–2973 (2000). 10737778 10.1073/pnas.97.7.2968 PMC16175

[11] R. Zwanzig, Nonequilibrium Statistical Mechanics (Oxford University Press, 2001).

[12] J. Wouters, V. Lucarini, Disentangling multi-level systems: Averaging, correlations and memory. J. Stat. Mech. Theory Exp. **2012**, P03003 (2012).

[13] J. Wouters, V. Lucarini, Multi-level dynamical systems: Connecting the Ruelle response theory and the Mori-Zwanzig approach. J. Stat. Phys. **151**, 850–860 (2013).

[14] R. Lam , Learning skillful medium-range global weather forecasting. Science **382**, 1416–1421 (2023). 37962497 10.1126/science.adi2336

[15] J. Pathak *et al*., A global data-driven high-resolution weather model using adaptive Fourier neural operators. arXiv [Preprint] (2022). https://arxiv.org/abs/2202.11214 (Accessed 30 March 2026).

[16] I. Price *et al*., Diffusion-based ensemble forecasting for medium-range weather. arXiv [Preprint] (2023). https://arxiv.org/abs/2312.15796 (Accessed 30 March 2026).

[17] D. Kochkov , Neural general circulation models for weather and climate. Nature **632**, 1060–1066 (2024). 39039241 10.1038/s41586-024-07744-y PMC11357988

[18] S. Rasp, M. S. Pritchard, P. Gentine, Deep learning to represent subgrid processes in climate models. Proc. Natl. Acad. Sci. U.S.A. **115**, 9684–9689 (2018). 30190437 10.1073/pnas.1810286115 PMC6166853

[19] N. D. Brenowitz, C. S. Bretherton, Spatially extended tests of a neural network parametrization trained by coarse-graining. J. Adv. Model. Earth Syst. **11**, 2728–2744 (2019).

[20] J. Yuval, P. A. O’Gorman, Stable machine-learning parameterization of subgrid processes for climate modeling at a range of resolutions. Nat. Commun. **11**, 3295 (2020). 32620769 10.1038/s41467-020-17142-3 PMC7335176

[21] D. J. Gagne, H. M. Christensen, A. C. Subramanian, A. H. Monahan, Machine learning for stochastic parameterization: Generative adversarial networks in the Lorenz ’96 model. J. Adv. Model. Earth Syst. **12**, e2019MS001896 (2020).

[22] L. Zanna, T. Bolton, Data-driven equation discovery of ocean mesoscale closures. Geophys. Res. Lett. **47**, e2020GL088376 (2020).

[23] A. P. Guillaumin, L. Zanna, Stochastic-deep learning parameterization of ocean momentum forcing. J. Adv. Model. Earth Syst. **13**, e2021MS002534 (2021).

[24] P. Perezhogin, L. Zanna, C. Fernandez-Granda, Generative data-driven approaches for stochastic subgrid parameterizations in an idealized ocean model. J. Adv. Model. Earth Syst. **15**, e2023MS003681 (2023).

[25] M. T. Brolly, Stochastic parameterization: The importance of nonlocality and memory. J. Adv. Model. Earth Syst. **17**, e2025MS005223 (2025).

[26] S Rasp, Coupled online learning as a way to tackle instabilities and biases in neural network parameterizations: General algorithms and Lorenz 96 case study (v1. 0). *Geosci. Model. Dev.* **13**, 2185–2196 (2020).

[27] D. Kochkov , Machine learning-accelerated computational fluid dynamics. Proc. Natl. Acad. Sci. U.S.A. **118**, e2101784118 (2021). 34006645 10.1073/pnas.2101784118 PMC8166023

[28] H. Frezat, J. Le Sommer, R. Fablet, G. Balarac, R. Lguensat, A posteriori learning for quasi-geostrophic turbulence parametrization. J. Adv. Model. Earth Syst. **14**, e2022MS003124 (2022).

[29] J. R. Maddison, Online learning in idealized ocean gyres. J. Adv. Model. Earth Syst. **18**, e2024MS004883 (2026).

[30] T. Gneiting, A. E. Raftery, Strictly proper scoring rules, prediction, and estimation. J. Am. Stat. Assoc. **102**, 359–378 (2007).

[31] I. J. Good, Rational decisions. J. R. Stat. Soc. Ser. B Methodol. **14**, 107–114 (1952).

[32] J. Bröcker, L. A. Smith, Scoring probabilistic forecasts: The importance of being proper. Weather. Forecast. **22**, 382–388 (2007).

[33] H. Du, Beyond strictly proper scoring rules: The importance of being local. Weather. Forecast. **36**, 457–468 (2021).

[34] C. A. T. Ferro, Fair scores for ensemble forecasts. Q. J. R. Meteorol. Soc. **140**, 1917–1923 (2014).

[35] K. Bi , Accurate medium-range global weather forecasting with 3d neural networks. Nature **619**, 533–538 (2023). 37407823 10.1038/s41586-023-06185-3 PMC10356604

[36] M. Chantry *et al*., “AIFS – ECMWF’s data-driven forecasting system” in *105th Annual AMS Meeting 2025* (American Meteorological Society, 2025), vol. 105, p. 449087.

[37] S. Allen, J. Ziegel, D. Ginsbourger, Assessing the calibration of multivariate probabilistic forecasts. Q. J. R. Meteorol. Soc. **150**, 1315–1335 (2024).

[38] M. T. Brolly, mtbrolly/qg_closure: Version at article submission. Zenodo. 10.5281/zenodo.19335604. Deposited 30 March 2026.

[39] M. T. Brolly, Trained neural networks for the paper “Stochasticity and probabilistic trajectory scoring are essential for data-driven closures of chaotic systems”. Zenodo. 10.5281/zenodo.19335706. Deposited 30 March 2026. PMC13534829 42636379

