# Supplementary material for: Stochasticity and probabilistic trajectory scoring are essential for data-driven closures of chaotic systems
Source: Proc Natl Acad Sci U S A. 2026 Aug 24;123(35):e2609143123. doi: 10.1073/pnas.2609143123 (PMC13534829; doi:10.1073/pnas.2609143123)
Supplement: Supplementary file 1 [file pnas.2609143123.sapp.pdf]

Supporting Information for

Stochasticity and probabilistic trajectory scoring are essential for data-driven closures of chaotic systems

Martin T. Brolly

Corresponding author: Martin T. Brolly.
E-mail: m.brolly@ed.ac.uk

This PDF file includes:

Supporting text
Fig. S1
Table S1
SI References

Supporting Information Text

Proofs of main-text results

Proof of Proposition 1. Let $\bar{\mu} := \mu \circ \overline{(\cdot)}^{-1}$ be the pushforward invariant measure on the resolved space. By construction, the trajectories of the model are generated by the recurrence $\widetilde{\boldsymbol{x}}_{n+k+1} = \boldsymbol{F}_{\boldsymbol{\theta}}(\widetilde{\boldsymbol{x}}_{n+k},\, \boldsymbol{\xi}_{n+k})$, starting from the exact initial condition $\widetilde{\boldsymbol{x}}_n = \overline{\boldsymbol{x}}_n$. Therefore, the prediction at lead time m can be written as a deterministic composition of the initial condition and the accumulated sequence of independent noise variables, which we denote as $\boldsymbol{\Xi}_{n,m} = (\boldsymbol{\xi}_n,\, \boldsymbol{\xi}_{n+1},\, \ldots,\, \boldsymbol{\xi}_{n+m-1})$, yielding

$$\widetilde{\boldsymbol{x}}_{n+m} = \boldsymbol{F}_{\boldsymbol{\theta}}^{(m)}(\overline{\boldsymbol{x}}_n,\, \boldsymbol{\Xi}_{n,m}).$$

We expand the mean-squared-error objective using the law of total expectation, conditioning on the resolved state $\overline{\boldsymbol{x}}_n \sim \bar{\mu}$ to obtain

$$\mathcal{J}_m(\boldsymbol{\theta}) = \mathbb{E}_{\bar{\mu}}\left[\mathbb{E}\left[\left\|\widetilde{\boldsymbol{x}}_{n+m} - \overline{\boldsymbol{x}}_{n+m}\right\|^2 \,\middle|\, \overline{\boldsymbol{x}}_n\right]\right].$$

For any fixed initial condition $\overline{\boldsymbol{x}}_n$, we apply the multivariate bias-variance identity to the inner conditional expectation, which gives

$$\begin{aligned}\mathbb{E}\left[\left\|\widetilde{\boldsymbol{x}}_{n+m} - \overline{\boldsymbol{x}}_{n+m}\right\|^2 \,\middle|\, \overline{\boldsymbol{x}}_n\right] &= \left\|\mathbb{E}\left[\widetilde{\boldsymbol{x}}_{n+m} \mid \overline{\boldsymbol{x}}_n\right] - \mathbb{E}\left[\overline{\boldsymbol{x}}_{n+m} \mid \overline{\boldsymbol{x}}_n\right]\right\|^2 \\ &\quad + \operatorname{Tr}\Big(\operatorname{Cov}\left(\widetilde{\boldsymbol{x}}_{n+m} \mid \overline{\boldsymbol{x}}_n\right)\Big) + \operatorname{Tr}\Big(\operatorname{Cov}\left(\overline{\boldsymbol{x}}_{n+m} \mid \overline{\boldsymbol{x}}_n\right)\Big) \\ &\quad - 2\operatorname{Tr}\Big(\operatorname{Cov}\left(\widetilde{\boldsymbol{x}}_{n+m},\, \overline{\boldsymbol{x}}_{n+m} \mid \overline{\boldsymbol{x}}_n\right)\Big).\end{aligned}$$

Conditioned on $\overline{\boldsymbol{x}}_n$, the surrogate prediction $\widetilde{\boldsymbol{x}}_{n+m}$ is a measurable function of $(\overline{\boldsymbol{x}}_n, \boldsymbol{\Xi}_{n,m})$ and hence its only source of randomness is the injected noise sequence $\boldsymbol{\Xi}_{n,m}$. By construction, $\boldsymbol{\Xi}_{n,m}$ is generated independently of the true system and, in particular, satisfies the conditional independence $\boldsymbol{\Xi}_{n,m} \perp\!\!\!\perp \boldsymbol{x}'_n \mid \overline{\boldsymbol{x}}_n$. Since the true future $\overline{\boldsymbol{x}}_{n+m}$ is a measurable function of $(\overline{\boldsymbol{x}}_n, \boldsymbol{x}'_n)$, it follows that $\widetilde{\boldsymbol{x}}_{n+m} \perp\!\!\!\perp \overline{\boldsymbol{x}}_{n+m} \mid \overline{\boldsymbol{x}}_n$, and therefore

$$\operatorname{Cov}\left(\widetilde{\boldsymbol{x}}_{n+m},\, \overline{\boldsymbol{x}}_{n+m} \mid \overline{\boldsymbol{x}}_n\right) = \boldsymbol{0}.$$

Taking the outer expectation over the coarse-grained invariant measure $\bar{\mu}$, the total loss separates into three non-negative terms:

$$\begin{aligned}\mathcal{J}_m(\boldsymbol{\theta}) &= \mathbb{E}_{\bar{\mu}}\left[\left\|\mathbb{E}\left[\widetilde{\boldsymbol{x}}_{n+m} \mid \overline{\boldsymbol{x}}_n\right] - \mathbb{E}\left[\overline{\boldsymbol{x}}_{n+m} \mid \overline{\boldsymbol{x}}_n\right]\right\|^2\right] && \text{[Conditional Bias]} \\ &\quad + \mathbb{E}_{\bar{\mu}}\left[\operatorname{Tr}\Big(\operatorname{Cov}\left(\widetilde{\boldsymbol{x}}_{n+m} \mid \overline{\boldsymbol{x}}_n\right)\Big)\right] && \text{[Predictive Variance]} \\ &\quad + \mathbb{E}_{\bar{\mu}}\left[\operatorname{Tr}\Big(\operatorname{Cov}\left(\overline{\boldsymbol{x}}_{n+m} \mid \overline{\boldsymbol{x}}_n\right)\Big)\right]. && \text{[Target Uncertainty]}\end{aligned}$$

The third term (Target Uncertainty) depends solely on the true dynamics and represents the irreducible error given the initial condition. Defining C_m as this term, it is independent of the model parameters $\boldsymbol{\theta}$, which establishes the exact decomposition stated in the proposition. Because the Conditional Bias and Predictive Variance terms are expectations of non-negative quantities, they act as non-negative additive penalties in the objective.

By assumption, the coarse-grained state decorrelates under the dynamics such that

$$\mathbb{E}\left[\overline{\boldsymbol{x}}_{n+m} \mid \overline{\boldsymbol{x}}_n\right] \xrightarrow{L^2(\bar{\mu})} \mathbb{E}_{\bar{\mu}}[\overline{\boldsymbol{x}}] \qquad \text{as } m \to \infty.$$

Therefore, in the exact decomposition above, the target conditional mean appearing in the conditional-bias term approaches the climatological mean at long lead times, while the predictive-variance term remains an additive non-negative penalty. Combined with the exact decomposition, this proves the proposition. □

Continuity and integrability assumptions used in Proposition 2. Throughout this section, let $\mathcal{P}_2$ denote the set of Borel probability measures on the resolved space with finite second moment. For each fixed parameter value $\boldsymbol{\theta}$ and lead time m, define

$$f_{m,\boldsymbol{\theta}}(\overline{\boldsymbol{x}}_n) := \mathbb{E}_{\overline{\boldsymbol{y}} \sim Q_m(\cdot \mid \overline{\boldsymbol{x}}_n)}\left[\mathcal{S}\left(\widetilde{\mathbb{P}}_{\boldsymbol{\theta},m}(\cdot \mid \overline{\boldsymbol{x}}_n), \overline{\boldsymbol{y}}\right)\right].$$

The continuity and integrability assumptions referenced in main-text Proposition 2 are the following:

1. The expected score functional
$$(P, Q) \mapsto \mathbb{E}_{\boldsymbol{y} \sim Q}\left[\mathcal{S}(P, \boldsymbol{y})\right]$$
is jointly continuous with respect to $W_2 \times W_2$ on $\mathcal{P}_2 \times \mathcal{P}_2$.

2. For each fixed $\boldsymbol{\theta}$, the sequence

$$\{f_{m,\boldsymbol{\theta}}(\overline{\boldsymbol{x}}_n)\}_{m\geq 1}$$

is uniformly integrable under $\bar{\mu}$.

Proof of Proposition 2. Let $\bar{\mu} := \mu \circ \overline{(\cdot)}^{-1}$ be the pushforward invariant measure on the resolved space, and use the notation introduced above. By assumption, the true lead-time conditional distributions satisfy

$$Q_m(\cdot \mid \overline{\boldsymbol{x}}_n) \xrightarrow{W_2} \bar{\mu}(\cdot)$$

for $\bar{\mu}$-almost every $\overline{\boldsymbol{x}}_n$, and the model predictive distributions admit a W_2 limit

$$\widetilde{\mathbb{P}}_{\boldsymbol{\theta},m}(\cdot \mid \overline{\boldsymbol{x}}_n) \xrightarrow{W_2} \widetilde{\mathbb{P}}_{\boldsymbol{\theta},\infty}(\cdot \mid \overline{\boldsymbol{x}}_n)$$

for $\bar{\mu}$-almost every $\overline{\boldsymbol{x}}_n$.

By joint continuity of the expected score functional with respect to $W_2 \times W_2$,

$$f_{m,\boldsymbol{\theta}}(\overline{\boldsymbol{x}}_n) \to f_{\infty,\boldsymbol{\theta}}(\overline{\boldsymbol{x}}_n) := \mathbb{E}_{\overline{\boldsymbol{y}}\sim\bar{\mu}}\left[\mathcal{S}\left(\widetilde{\mathbb{P}}_{\boldsymbol{\theta},\infty}(\cdot \mid \overline{\boldsymbol{x}}_n), \overline{\boldsymbol{y}}\right)\right]$$

for $\bar{\mu}$-almost every $\overline{\boldsymbol{x}}_n$.

Because the sequence $\{f_{m,\boldsymbol{\theta}}(\overline{\boldsymbol{x}}_n)\}_m$ is uniformly integrable under $\bar{\mu}$ for each fixed $\boldsymbol{\theta}$, we may pass the limit through the outer expectation, yielding

$$\lim_{m\to\infty} \mathcal{L}_m(\boldsymbol{\theta}) = \mathbb{E}_{\overline{\boldsymbol{x}}_n}\left[f_{\infty,\boldsymbol{\theta}}(\overline{\boldsymbol{x}}_n)\right].$$

Every strictly proper scoring rule $\mathcal{S}$ induces a non-negative divergence

$$d_{\mathcal{S}}(P,Q) := \mathbb{E}_{\overline{\boldsymbol{y}}\sim Q}\left[\mathcal{S}(P,\overline{\boldsymbol{y}})\right] - \mathbb{E}_{\overline{\boldsymbol{y}}\sim Q}\left[\mathcal{S}(Q,\overline{\boldsymbol{y}})\right] \geq 0,$$

with equality if and only if $P = Q$. Rearranging gives

$$\mathbb{E}_{\overline{\boldsymbol{y}}\sim Q}\left[\mathcal{S}(P,\overline{\boldsymbol{y}})\right] = d_{\mathcal{S}}(P,Q) + \mathbb{E}_{\overline{\boldsymbol{y}}\sim Q}\left[\mathcal{S}(Q,\overline{\boldsymbol{y}})\right].$$

Applying this identity with

$$P = \widetilde{\mathbb{P}}_{\boldsymbol{\theta},\infty}(\cdot \mid \overline{\boldsymbol{x}}_n), \qquad Q = \bar{\mu},$$

we obtain

$$\lim_{m\to\infty} \mathcal{L}_m(\boldsymbol{\theta}) = \mathbb{E}_{\overline{\boldsymbol{x}}_n}\left[d_{\mathcal{S}}\left(\widetilde{\mathbb{P}}_{\boldsymbol{\theta},\infty}(\cdot \mid \overline{\boldsymbol{x}}_n), \bar{\mu}\right)\right] + \mathbb{E}_{\overline{\boldsymbol{y}}\sim\bar{\mu}}\left[\mathcal{S}(\bar{\mu},\overline{\boldsymbol{y}})\right].$$

The second term depends only on the physical system and the choice of scoring rule, so it is independent of the model parameters $\boldsymbol{\theta}$. Thus the parameter-dependent part of the asymptotic objective is exactly the expected divergence between the model's long-lead predictive distribution and the invariant measure. □

Supplementary theoretical result

The degeneracy identified in main-text Proposition 1 is not specific to mean-squared error. It is a structural feature of any objective that evaluates forecasts only through pointwise discrepancies against realized targets, without rewarding predictive variability.

Formal setting. Let the resolved state space $(\overline{\mathcal{X}}, d)$ be a Polish metric space, fix a reference point $x_0 \in \overline{\mathcal{X}}$, and let $\mathcal{P}_p(\overline{\mathcal{X}})$ denote the space of Borel probability measures ν on $\overline{\mathcal{X}}$ such that

$$\int_{\overline{\mathcal{X}}} d(x,x_0)^p \, \mathrm{d}\nu(x) < \infty.$$

Let $\bar{\mu} := \mu \circ \overline{(\cdot)}^{-1} \in \mathcal{P}_p(\overline{\mathcal{X}})$ be the pushforward invariant measure on the resolved space, and assume $\overline{\boldsymbol{x}}_n \sim \bar{\mu}$.

We adopt the Polish metric space setting to make clear that the result is not a peculiarity of Euclidean state vectors or the standard norm. The argument depends only on the metric geometry of the resolved space, and therefore applies more generally than a coordinate-based formulation. It also avoids the bookkeeping that comes with fixing a particular coordinate system.

Definition 1 (Pointwise losses)**.** *Let*

$$\ell : \overline{\mathcal{X}} \times \overline{\mathcal{X}} \to \mathbb{R}$$

be a continuous bivariate loss function such that for all $\nu \in \mathcal{P}_p(\overline{\mathcal{X}})$ and all $\overline{\boldsymbol{x}} \in \overline{\mathcal{X}}$,

$$\int_{\overline{\mathcal{X}}} \left|\ell(\widetilde{\boldsymbol{x}}, \overline{\boldsymbol{x}})\right| \mathrm{d}\nu(\widetilde{\boldsymbol{x}}) < \infty.$$

The associated pointwise loss is

$$\Lambda(\nu, \overline{\boldsymbol{x}}) := \mathbb{E}_{\widetilde{\boldsymbol{x}}\sim\nu}\left[\ell(\widetilde{\boldsymbol{x}}, \overline{\boldsymbol{x}})\right].$$

For a deterministic forecast $\widetilde{\boldsymbol{x}}_d$, this reduces to $\Lambda(\delta_{\widetilde{\boldsymbol{x}}_d}, \overline{\boldsymbol{x}}) = \ell(\widetilde{\boldsymbol{x}}_d, \overline{\boldsymbol{x}})$, where $\delta_{\widetilde{\boldsymbol{x}}_d}$ denotes the Dirac measure at $\widetilde{\boldsymbol{x}}_d$.

Supplementary Proposition 1 (Degeneracy of pointwise losses)**.** *Assume that for $\bar{\mu}$-almost every $\overline{\boldsymbol{x}}_n$, the true conditional measure $Q_m(\cdot \mid \overline{\boldsymbol{x}}_n) := \mathbb{P}(\overline{\boldsymbol{x}}_{n+m} \in \cdot \mid \overline{\boldsymbol{x}}_n)$ converges in W_p to the invariant measure:*

$$Q_m(\cdot \mid \overline{\boldsymbol{x}}_n) \xrightarrow{W_p} \bar{\mu}(\cdot) \qquad \text{as } m \to \infty.$$

Consider a Markovian model $\widetilde{\boldsymbol{x}}_{n+k+1} = \boldsymbol{F}_{\boldsymbol{\theta}}(\widetilde{\boldsymbol{x}}_{n+k}, \boldsymbol{\xi}_{n+k})$, with $\widetilde{\boldsymbol{x}}_n = \overline{\boldsymbol{x}}_n$, and let $\widetilde{\mathbb{P}}_{\boldsymbol{\theta},m}(\cdot \mid \overline{\boldsymbol{x}}_n) \in \mathcal{P}_p(\overline{\mathcal{X}})$ denote its m-step conditional predictive measure. Assume that for $\bar{\mu}$-almost every $\overline{\boldsymbol{x}}_n$,

$$\widetilde{\mathbb{P}}_{\boldsymbol{\theta},m}(\cdot \mid \overline{\boldsymbol{x}}_n) \xrightarrow{W_p} \widetilde{\mathbb{P}}_{\boldsymbol{\theta},\infty}(\cdot \mid \overline{\boldsymbol{x}}_n).$$

Let ℓ be a continuous bivariate loss satisfying the growth bound

$$|\ell(\widetilde{\boldsymbol{y}}, \overline{\boldsymbol{y}})| \le C\Big(1 + d(\widetilde{\boldsymbol{y}}, x_0)^p + d(\overline{\boldsymbol{y}}, x_0)^p\Big)$$

for some constant $C > 0$ and all $\widetilde{\boldsymbol{y}}, \overline{\boldsymbol{y}} \in \overline{\mathcal{X}}$, and assume that the induced expected loss functional

$$(P, Q) \mapsto \int_{\overline{\mathcal{X}}} \int_{\overline{\mathcal{X}}} \ell(\widetilde{\boldsymbol{y}}, \overline{\boldsymbol{y}})\, \mathrm{d}P(\widetilde{\boldsymbol{y}})\, \mathrm{d}Q(\overline{\boldsymbol{y}})$$

is jointly continuous with respect to $W_p \times W_p$. Define the climatological risk $R(\boldsymbol{c}) := \mathbb{E}_{\overline{\boldsymbol{y}} \sim \bar{\mu}}[\ell(\boldsymbol{c}, \overline{\boldsymbol{y}})]$, and assume that R has a unique global minimizer $\boldsymbol{c}^* \in \overline{\mathcal{X}}$.

For each lead time m, define the conditional expected loss:

$$g_{m,\boldsymbol{\theta}}(\overline{\boldsymbol{x}}_n) := \int_{\overline{\mathcal{X}}} \int_{\overline{\mathcal{X}}} \ell(\widetilde{\boldsymbol{y}}, \overline{\boldsymbol{y}})\, \mathrm{d}\widetilde{\mathbb{P}}_{\boldsymbol{\theta},m}(\widetilde{\boldsymbol{y}} \mid \overline{\boldsymbol{x}}_n)\, \mathrm{d}Q_m(\overline{\boldsymbol{y}} \mid \overline{\boldsymbol{x}}_n).$$

The corresponding expected loss is $\mathcal{J}^{\ell}_m(\boldsymbol{\theta}) = \mathbb{E}_{\overline{\boldsymbol{x}}_n}[g_{m,\boldsymbol{\theta}}(\overline{\boldsymbol{x}}_n)]$. Assume additionally that, for each fixed $\boldsymbol{\theta}$, the sequence $\{g_{m,\boldsymbol{\theta}}(\overline{\boldsymbol{x}}_n)\}_{m \ge 1}$ is uniformly integrable under $\bar{\mu}$. Then

$$\lim_{m \to \infty} \mathcal{J}^{\ell}_m(\boldsymbol{\theta}) = \mathbb{E}_{\overline{\boldsymbol{x}}_n}\left[\int_{\overline{\mathcal{X}}} R(\widetilde{\boldsymbol{y}})\, \mathrm{d}\widetilde{\mathbb{P}}_{\boldsymbol{\theta},\infty}(\widetilde{\boldsymbol{y}} \mid \overline{\boldsymbol{x}}_n)\right].$$

In particular,

$$\lim_{m \to \infty} \mathcal{J}^{\ell}_m(\boldsymbol{\theta}) \ge R(\boldsymbol{c}^*),$$

with equality if and only if

$$\widetilde{\mathbb{P}}_{\boldsymbol{\theta},\infty}(\cdot \mid \overline{\boldsymbol{x}}_n) = \delta_{\boldsymbol{c}^*}(\cdot) \qquad \text{for } \bar{\mu}\text{-almost every } \overline{\boldsymbol{x}}_n.$$

Thus, optimizing any pointwise loss over long lead times forces collapse onto a deterministic climatological risk minimizer.

Proof of Supplementary Proposition 1. Conditioning on the initial state $\overline{\boldsymbol{x}}_n$, the conditional expected loss is given by

$$g_{m,\boldsymbol{\theta}}(\overline{\boldsymbol{x}}_n) := \int_{\overline{\mathcal{X}}} \int_{\overline{\mathcal{X}}} \ell(\widetilde{\boldsymbol{y}}, \overline{\boldsymbol{y}})\, \mathrm{d}\widetilde{\mathbb{P}}_{\boldsymbol{\theta},m}(\widetilde{\boldsymbol{y}} \mid \overline{\boldsymbol{x}}_n)\, \mathrm{d}Q_m(\overline{\boldsymbol{y}} \mid \overline{\boldsymbol{x}}_n).$$

The overall objective is given by $\mathcal{J}^{\ell}_m(\boldsymbol{\theta}) = \mathbb{E}_{\overline{\boldsymbol{x}}_n}[g_{m,\boldsymbol{\theta}}(\overline{\boldsymbol{x}}_n)]$.

By assumption, for $\bar{\mu}$-almost every $\overline{\boldsymbol{x}}_n$, $Q_m \xrightarrow{W_p} \bar{\mu}$ and $\widetilde{\mathbb{P}}_{\boldsymbol{\theta},m} \xrightarrow{W_p} \widetilde{\mathbb{P}}_{\boldsymbol{\theta},\infty}$. Joint continuity of the expected loss functional yields $g_{m,\boldsymbol{\theta}}(\overline{\boldsymbol{x}}_n) \to g_{\infty,\boldsymbol{\theta}}(\overline{\boldsymbol{x}}_n)$, where

$$g_{\infty,\boldsymbol{\theta}}(\overline{\boldsymbol{x}}_n) := \int_{\overline{\mathcal{X}}} \int_{\overline{\mathcal{X}}} \ell(\widetilde{\boldsymbol{y}}, \overline{\boldsymbol{y}})\, \mathrm{d}\widetilde{\mathbb{P}}_{\boldsymbol{\theta},\infty}(\widetilde{\boldsymbol{y}} \mid \overline{\boldsymbol{x}}_n)\, \mathrm{d}\bar{\mu}(\overline{\boldsymbol{y}}).$$

Since $\{g_{m,\boldsymbol{\theta}}(\overline{\boldsymbol{x}}_n)\}_{m \ge 1}$ is uniformly integrable under $\bar{\mu}$, we may pass the limit through the outer expectation: $\lim_{m \to \infty} \mathcal{J}^{\ell}_m(\boldsymbol{\theta}) = \mathbb{E}_{\overline{\boldsymbol{x}}_n}[g_{\infty,\boldsymbol{\theta}}(\overline{\boldsymbol{x}}_n)]$. Now define the climatological risk $R(\widetilde{\boldsymbol{y}}) := \int_{\overline{\mathcal{X}}} \ell(\widetilde{\boldsymbol{y}}, \overline{\boldsymbol{y}})\, \mathrm{d}\bar{\mu}(\overline{\boldsymbol{y}})$. By Fubini's theorem and the growth condition on ℓ,

$$g_{\infty,\boldsymbol{\theta}}(\overline{\boldsymbol{x}}_n) = \int_{\overline{\mathcal{X}}} R(\widetilde{\boldsymbol{y}})\, \mathrm{d}\widetilde{\mathbb{P}}_{\boldsymbol{\theta},\infty}(\widetilde{\boldsymbol{y}} \mid \overline{\boldsymbol{x}}_n),$$

and hence

$$\lim_{m \to \infty} \mathcal{J}^{\ell}_m(\boldsymbol{\theta}) = \mathbb{E}_{\overline{\boldsymbol{x}}_n}\left[\int_{\overline{\mathcal{X}}} R(\widetilde{\boldsymbol{y}})\, \mathrm{d}\widetilde{\mathbb{P}}_{\boldsymbol{\theta},\infty}(\widetilde{\boldsymbol{y}} \mid \overline{\boldsymbol{x}}_n)\right].$$

Because R has a unique global minimizer $\boldsymbol{c}^*$, $R(\widetilde{\boldsymbol{y}}) \ge R(\boldsymbol{c}^*)$ for all $\widetilde{\boldsymbol{y}} \in \overline{\mathcal{X}}$, with equality if and only if $\widetilde{\boldsymbol{y}} = \boldsymbol{c}^*$. Therefore,

$$\lim_{m \to \infty} \mathcal{J}^{\ell}_m(\boldsymbol{\theta}) \ge \mathbb{E}_{\overline{\boldsymbol{x}}_n}\left[\int_{\overline{\mathcal{X}}} R(\boldsymbol{c}^*)\, \mathrm{d}\widetilde{\mathbb{P}}_{\boldsymbol{\theta},\infty}(\widetilde{\boldsymbol{y}} \mid \overline{\boldsymbol{x}}_n)\right] = R(\boldsymbol{c}^*).$$

Thus $R(\boldsymbol{c}^*)$ is the minimum possible value of the asymptotic objective. Equality holds if and only if

$$\mathbb{E}_{\overline{\boldsymbol{x}}_n}\left[\int_{\overline{\mathcal{X}}}\left(R(\widetilde{\boldsymbol{y}})-R(\boldsymbol{c}^*)\right)\mathrm{d}\widetilde{\mathbb{P}}_{\boldsymbol{\theta},\infty}(\widetilde{\boldsymbol{y}}\mid\overline{\boldsymbol{x}}_n)\right]=0.$$

The integrand is nonnegative, so this occurs if and only if the inner integral is zero for $\bar{\mu}$-almost every $\overline{\boldsymbol{x}}_n$. Since $R(\widetilde{\boldsymbol{y}})-R(\boldsymbol{c}^*)>0$ for all $\widetilde{\boldsymbol{y}}\neq\boldsymbol{c}^*$, this is equivalent to $\widetilde{\mathbb{P}}_{\boldsymbol{\theta},\infty}(\cdot\mid\overline{\boldsymbol{x}}_n)=\delta_{\boldsymbol{c}^*}(\cdot)$ for $\bar{\mu}$-almost every $\overline{\boldsymbol{x}}_n$. □

Remark. Supplementary Proposition 1 applies to a broad class of losses. In particular, it covers deterministic hybrid objectives that augment physical-space mismatch with penalties on transformed features of the state. For the turbulence setting considered in this paper, a natural example is a loss that combines a physical-space error with a discrepancy between Fourier coefficient amplitudes of the forecast and target fields. Provided the resulting bivariate loss satisfies the continuity and growth assumptions above, it remains a pointwise loss in the sense of Definition 1: it compares a single forecast state with a single realized target state through a deterministic discrepancy. The proposition therefore still applies. Such modifications may change the induced climatological risk field, and may favor deterministic states with more realistic spectral structure, but they do not remove the underlying long-lead degeneracy: the asymptotic optimum is still attained only through collapse onto a deterministic risk minimizer, rather than through a nondegenerate predictive law.

Quasi-Geostrophic Turbulence Model Details

We consider a canonical idealized model of geophysical turbulence known as the Phillips model (1, 2). In this two-layer model the quasi-geostrophic potential vorticity (PV) Q is defined for each layer

$$Q_1=\boldsymbol{\nabla}^2\Psi_1+\beta y+\frac{k_d^2}{1+\frac{H_1}{H_2}}(\Psi_2-\Psi_1), \quad [1a]$$

$$Q_2=\boldsymbol{\nabla}^2\Psi_2+\beta y+\frac{H_1}{H_2}\frac{k_d^2}{1+\frac{H_1}{H_2}}(\Psi_1-\Psi_2). \quad [1b]$$

The parameter β arises from the linear approximation of the Coriolis parameter $f=f_0+\beta y$, H_1 and H_2 are the heights of the upper and lower layers, respectively, and $k_d^2=\frac{f_0^2}{g'}\frac{H_1+H_2}{H_1H_2}$, where $g'=g(\rho_2-\rho_1)/\rho_1$ is the reduced gravity. Up to the effects of friction, Q is materially conserved, so that in the presence of linear bottom friction and small-scale dissipation (denoted ssd), we have

$$\frac{\partial Q_1}{\partial t}+J(\Psi_1,\,Q_1)=\mathrm{ssd}, \quad [2a]$$

$$\frac{\partial Q_2}{\partial t}+J(\Psi_2,\,Q_2)=\mathrm{ssd}-\gamma\boldsymbol{\nabla}^2\Psi_2. \quad [2b]$$

We force the system by imposing a base state with vertical shear that is zonal and horizontally uniform. This choice can be motivated physically by either a meridional temperature gradient or zonal wind forcing. In particular, let the imposed mean velocity be $\overline{\boldsymbol{U}}_1=(\overline{U}_1,\,0)$ and $\overline{\boldsymbol{U}}_2=(0,\,0)$. Equivalently, let

$$\Psi_1(x,\,y,\,t)=-\overline{U}_1y+\psi_1(x,\,y,\,t), \quad [3a]$$

$$\Psi_2(x,\,y,\,t)=\psi_2(x,\,y,\,t), \quad [3b]$$

define the stream function anomaly ψ. Upon substituting Eq. (3) into Eq. (2) and defining the eddy PV

$$q_1=\boldsymbol{\nabla}^2\psi_1+\frac{k_d^2}{1+\frac{H_1}{H_2}}(\psi_2-\psi_1), \quad [4a]$$

$$q_2=\boldsymbol{\nabla}^2\psi_2+\frac{H_1}{H_2}\frac{k_d^2}{1+\frac{H_1}{H_2}}(\psi_1-\psi_2), \quad [4b]$$

we find

$$\frac{\partial q_1}{\partial t}+J(\psi_1,\,q_1)+\beta_1\frac{\partial\psi_1}{\partial x}+\overline{U}_1\frac{\partial q_1}{\partial x}=\mathrm{ssd}, \quad [5a]$$

$$\frac{\partial q_2}{\partial t}+J(\psi_2,\,q_2)+\beta_2\frac{\partial\psi_2}{\partial x}=\mathrm{ssd}-\gamma\boldsymbol{\nabla}^2\psi_2, \quad [5b]$$

with $(\beta_1,\,\beta_2)=\left(\beta+\frac{k_d^2}{1+\frac{H_1}{H_2}}\overline{U}_1,\,\beta-\frac{H_1}{H_2}\frac{k_d^2}{1+\frac{H_1}{H_2}}\overline{U}_1\right)$. Note from Eq. (1), Eq. (3) and Eq. (4), that the (total) PV in each layer is

$$Q_i=q_i+\beta_i y.$$

We solve Eq. (5) on a rectangular domain with doubly periodic PV anomaly. The model is implemented in JAX using a Fourier pseudospectral method and third-order Adams–Bashforth time stepping. The small-scale dissipation operator, ssd, is defined in the Fourier domain as a highly scale-selective low-pass filter, as first implemented by (3). Dealiasing is performed approximately, using the Fourier smoothing technique of (4), which retains significantly more small-scale energy than approximate dealiasing with the popular 2/3 Fourier truncation technique, but benefits from the same computational speed-up with respect to exact 3/2 dealiasing. In practice, the influence of the Fourier smoothing scheme is negligible, since the small-scale dissipation operator is already highly effective in controlling aliasing error.

Depending on the configuration of the model parameters, the resulting dynamics are characterized by the presence of coherent vortices and/or zonal jets (see e.g. 5). The model can also be configured to resemble more the dynamics of the ocean or the atmosphere. Here we adopt a configuration resembling midlatitude ocean dynamics, in which zonal jets are prominent. Physical parameter values are shown in Table S1 — these are the same as the "jet configuration" used by (6).

Neural Network Architecture and Training Details

State representation and closure architecture. In the quasi-geostrophic example, the resolved state at time step n is the stacked pair of coarse-grid potential-vorticity-anomaly fields in the two layers,

$$\overline{\boldsymbol{x}}_n = \big(\overline{q}_1(\cdot, t_n),\, \overline{q}_2(\cdot, t_n)\big),$$

represented in physical space on the coarse grid. The closure predicts the unresolved model-error field

$$\widetilde{\boldsymbol{m}}_n = \boldsymbol{G}_{\boldsymbol{\theta}}(\widetilde{\boldsymbol{x}}_n, \boldsymbol{\xi}_n),$$

where $\boldsymbol{\xi}_n$ is an independent standard Gaussian random field with the same spatial dimensions as the resolved state. Deterministic closures are obtained by removing the dependence on $\boldsymbol{\xi}_n$.

The map $\boldsymbol{G}_{\boldsymbol{\theta}}$ is implemented as a fully convolutional neural network with six convolutional layers, periodic padding, and width-5 filters. Each hidden layer is followed by batch normalization and a ReLU activation. In addition, each hidden layer is mapped to a two-channel output by a width-1 convolution, and these residual outputs are summed to form the final prediction. This architecture allows corrections from multiple depths of the network to contribute directly to the predicted model error. To enforce basic physical structure, the predicted model-error field is constrained to have zero spatial mean by setting the zero Fourier mode to zero. A high-pass spectral filter $\mathcal{G}(k_x, k_y) \propto k_x^2 + k_y^2$ is then applied to suppress spurious low-wavenumber energy. The resulting network contains 622,488 trainable parameters.

Data generation and coarse-graining. Training and validation data were generated from long integrations of the high-resolution reference model. Starting from a random initial condition, the model was first integrated for 100 years to reach approximate statistical stationarity. A further 100 years of simulation were then used to generate training data by saving the two-layer PV-anomaly field every 2 hours, matching the coarse-model time step and yielding 438,000 consecutive snapshots. An independent 100-year integration was used to generate an equally large validation dataset.

At each saved time, the fine-grid state is the stacked pair of high-resolution PV-anomaly fields. The resolved state is obtained by applying a coarse-graining operator that combines spectral truncation with the same low-pass filter used for small-scale dissipation in the coarse model, following (6). Thus, both the training data and the learned closure are defined on coarse-grid PV-anomaly fields in physical space, even though coarse-graining and numerical time stepping employ spectral operations. Figure S1 illustrates the effect of spectral truncation and subsequent filtering on representative PV-anomaly snapshots. The dominant large-scale flow features, including coherent zonal jets and mesoscale eddies, are retained by this procedure, although they are not fully resolved on the coarse grid.

Training objective, optimization, and curriculum learning. Closures were calibrated online using the energy score, which is a strictly proper scoring rule for ensemble forecasts. For a training window of length w, the model is initialized from the true resolved state and integrated forward for w coarse-model time steps. Parameters $\boldsymbol{\theta}$ are optimized to minimize the empirical mean energy score averaged over lead times within the window. For stochastic closures, this objective is estimated from ensembles of model realizations; for deterministic closures, the corresponding deterministic reduction of the energy score is used.

Optimization employed AdamW together with a cosine-decay learning-rate schedule with warm-up. Training used mini-batches of $B = 4$ trajectory windows and an ensemble size of $S = 4$ to obtain unbiased gradient estimates for the stochastic objective. To stabilize optimization over long horizons, training followed a 29-phase curriculum in which the window length was progressively increased from one coarse-model time step (2 hours) to 1200 time steps (100 days), with one epoch of training per phase. The independent validation dataset was used only for evaluation and not for parameter updates.

Parameter	Symbol	Value
Horizontal domain size	L_x, L_y	$1000\,\mathrm{km}$, $1000\,\mathrm{km}$
Heights of layers 1 and 2	H_1, H_2	$500\,\mathrm{m}$, $5000\,\mathrm{m}$
Mean upper layer zonal velocity	$\overline{U}_1$	$0.025\,\mathrm{m\,s^{-1}}$
Meridional gradient of the Coriolis parameter	β	$10^{-11}\,\mathrm{s^{-1}\,m^{-1}}$
Rate of bottom layer friction	γ	$7 \times 10^{-8}\,\mathrm{s^{-1}}$
First Rossby radius of deformation	$r_d = k_d^{-1}$	$15\,\mathrm{km}$
Coarse (fine) horizontal resolution	$n_x = n_y$	64 (1024)
Coarse (fine) time step	δt	2 hours (15 minutes)

Table S1. Physical parameters of the quasi-geostrophic turbulence model.

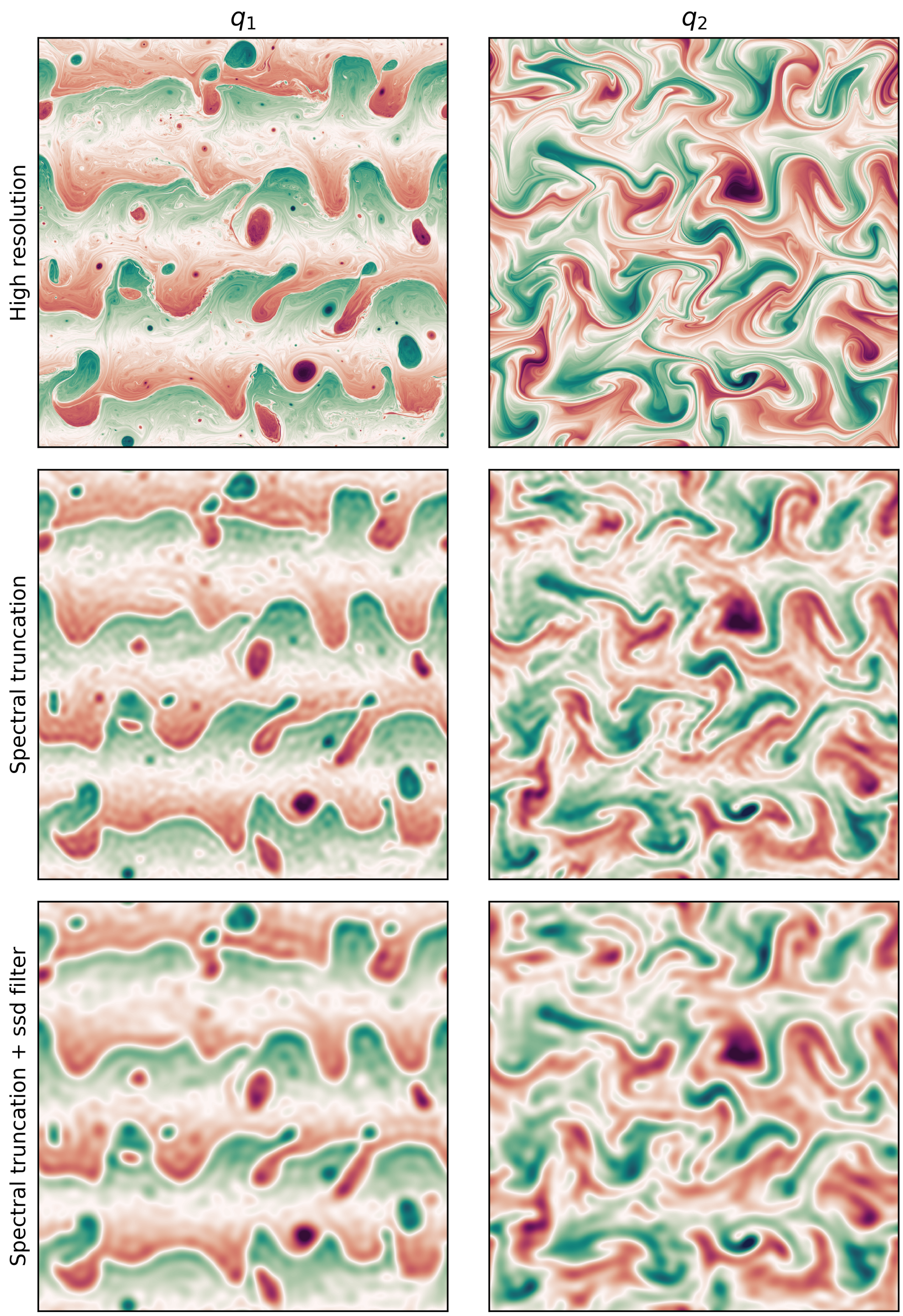

Fig. S1. Coarse-graining procedure: snapshots of PV anomaly in the upper layer (left) and lower layer (right) at high resolution (top row), after spectral truncation (middle row), and after spectral truncation followed by application of the low-pass filter used for small-scale dissipation in Eq. (5) (bottom row).

References

1. NA Phillips, Energy transformations and meridional circulations associated with simple baroclinic waves in a two-level, quasi-geostrophic model. *Tellus* **6**, 274–286 (1954).
2. GK Vallis, *Atmospheric and Oceanic Fluid Dynamics: Fundamentals and Large-Scale Circulation*. (Cambridge University Press), 2 edition, p. 171–212 (2017).
3. JH LaCasce, Ph.D. thesis (Massachusetts Institute of Technology) (1996).
4. TY Hou, R Li, Computing nearly singular solutions using pseudo-spectral methods. *J. Comput. Phys.* **226**, 379–397 (2007).
5. P Berloff, I Kamenkovich, *Dynamics of Baroclinic Multiple Zonal Jets*. (Cambridge University Press), p. 292–306 (2019).
6. A Ross, Z Li, P Perezhogin, C Fernandez-Granda, L Zanna, Benchmarking of machine learning ocean subgrid parameterizations in an idealized model. *J. Adv. Model. Earth Syst.* **15** (2023).
